# Supplementary material for: The two-component system ChvGI maintains cell envelope homeostasis in Caulobacter crescentus
Source: PLoS Genet. 2022 Dec 8;18(12):e1010465. doi: 10.1371/journal.pgen.1010465 (PMC9731502; doi:10.1371/journal.pgen.1010465)
Supplement: S6 Fig — (PDF) [file pgen.1010465.s006.pdf]

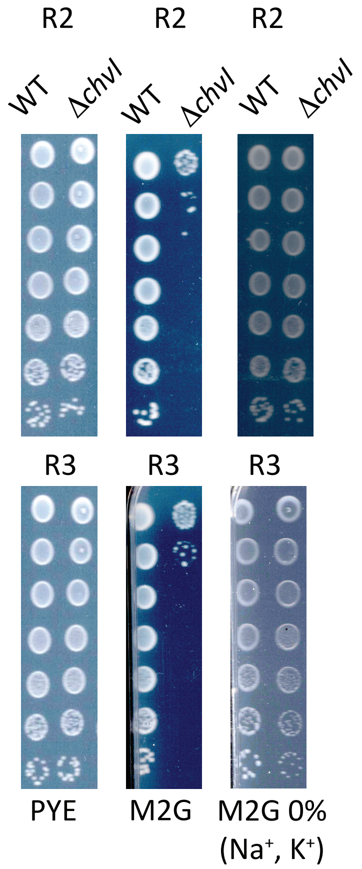

Replicates Figure 1.

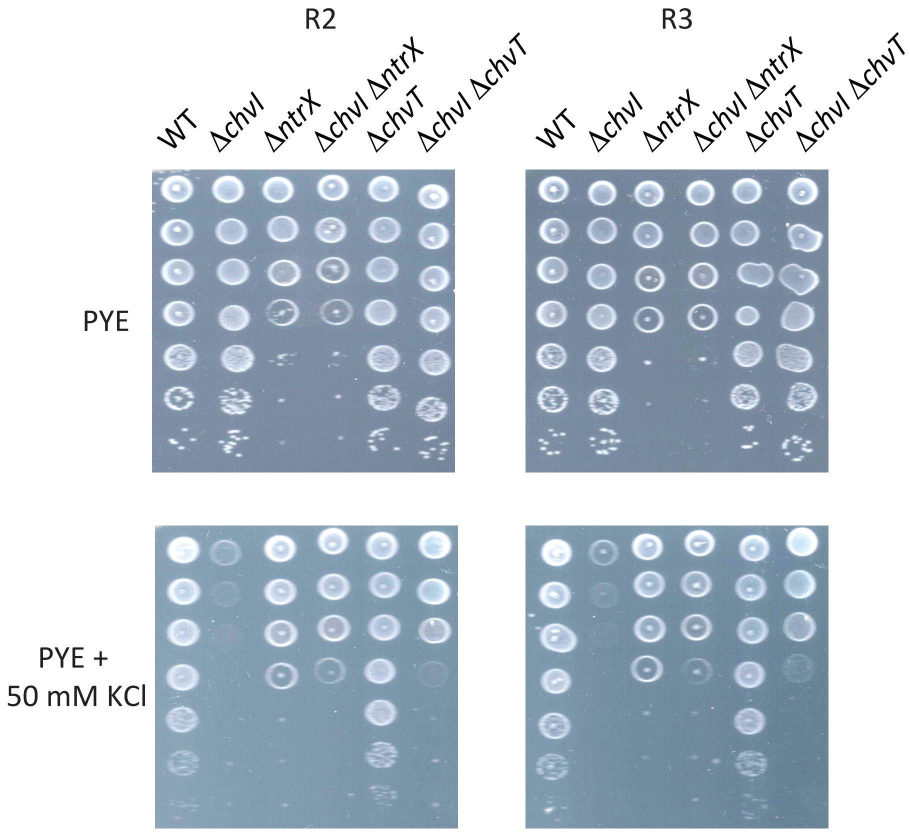

Replicates Figure 2.

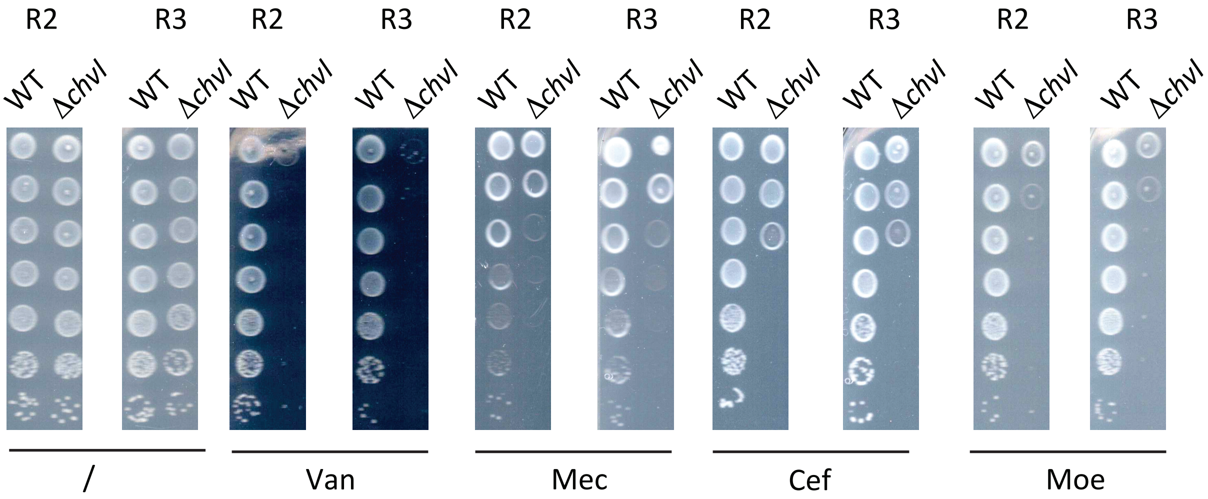

Replicates Figure 5E.

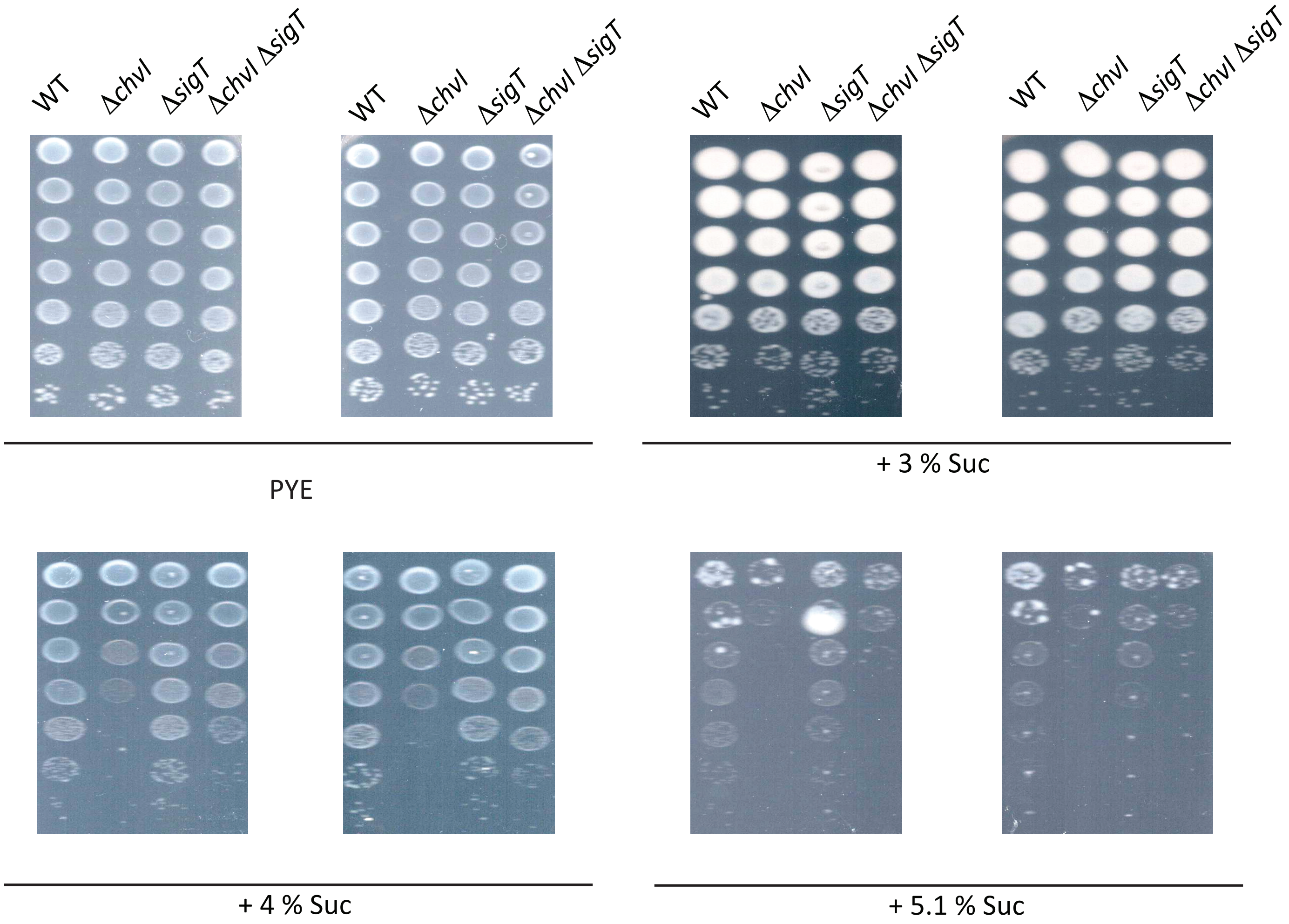

Replicates Figure S3B. Continues in next page

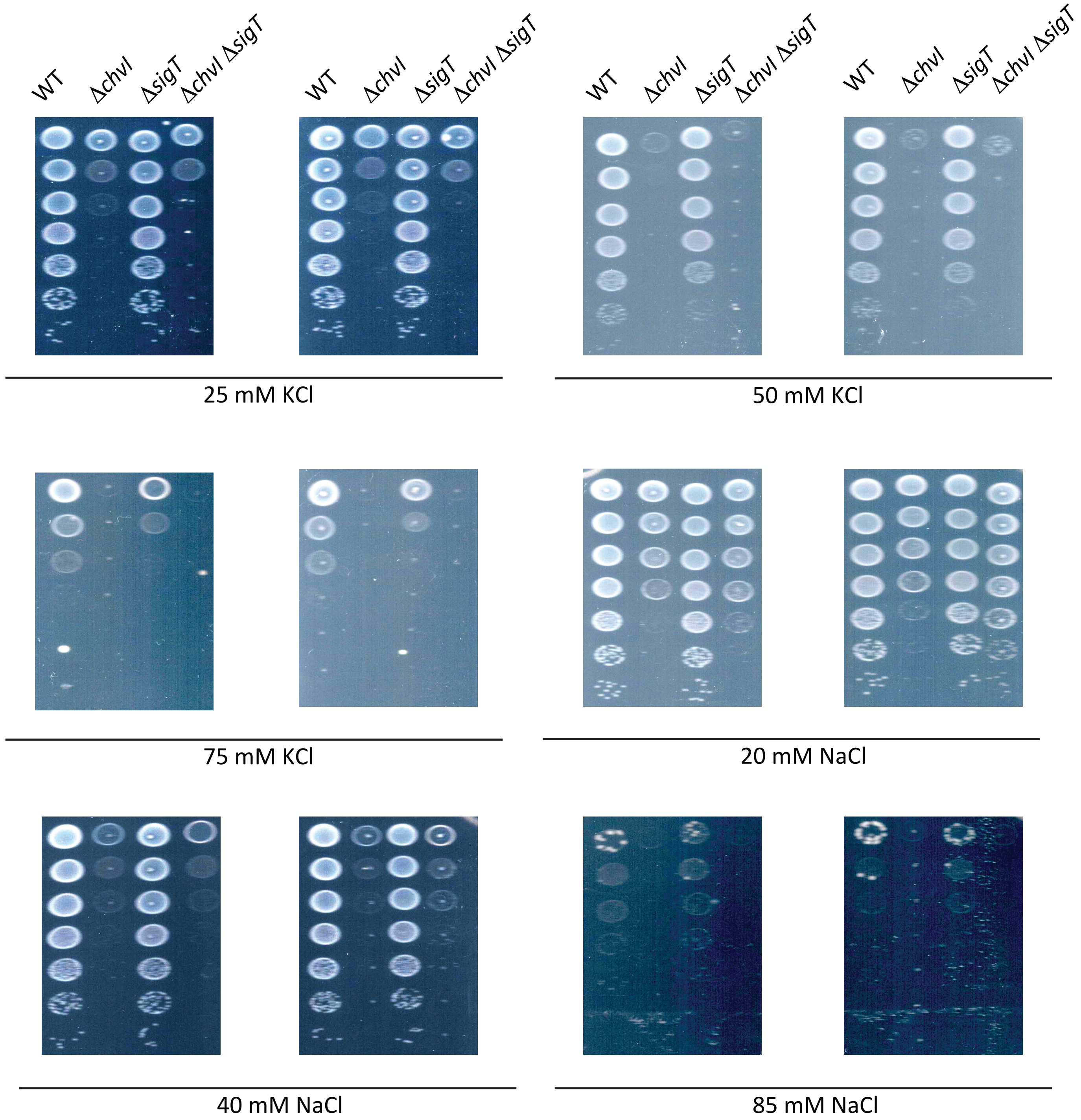

Replicates Figure S3B. Ends here

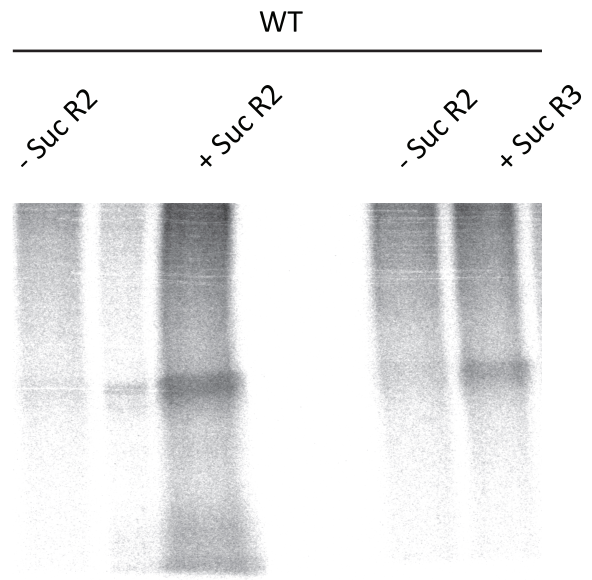

Replicates Figure 4C.

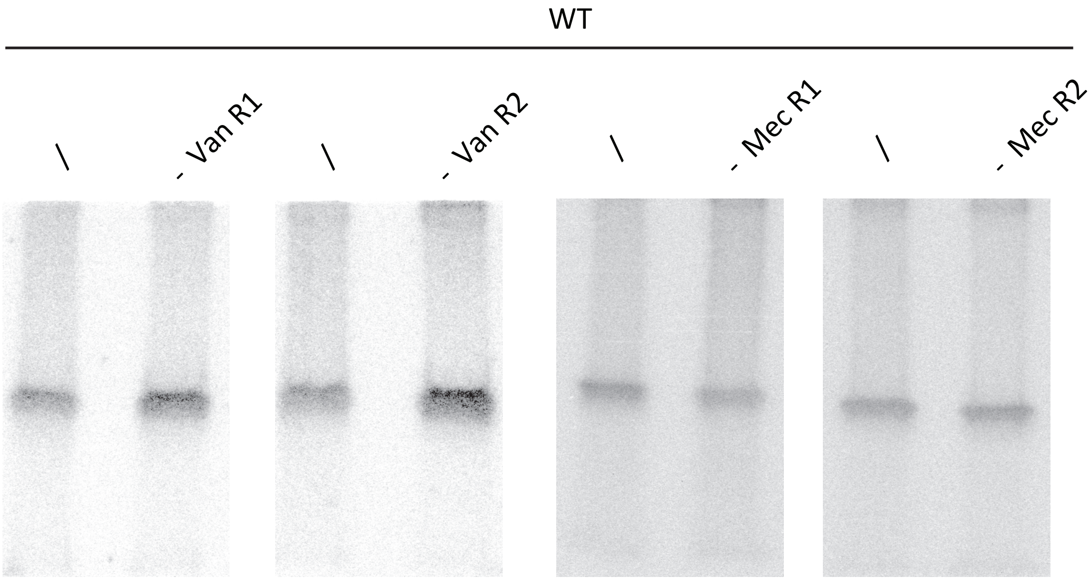

Replicates Figure 5F.

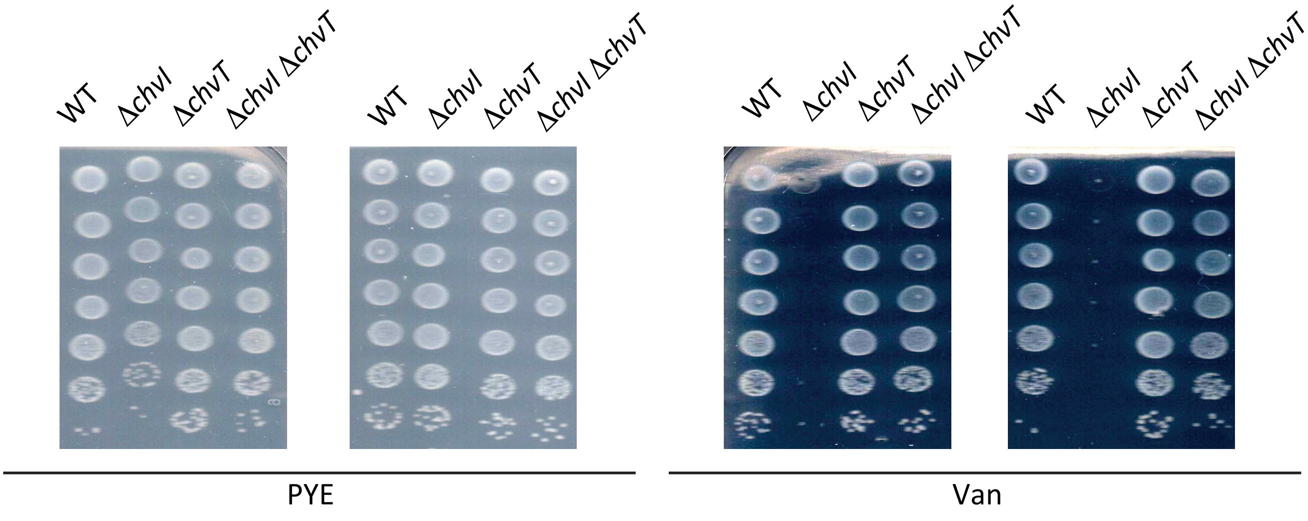

Replicates Figure 4C.

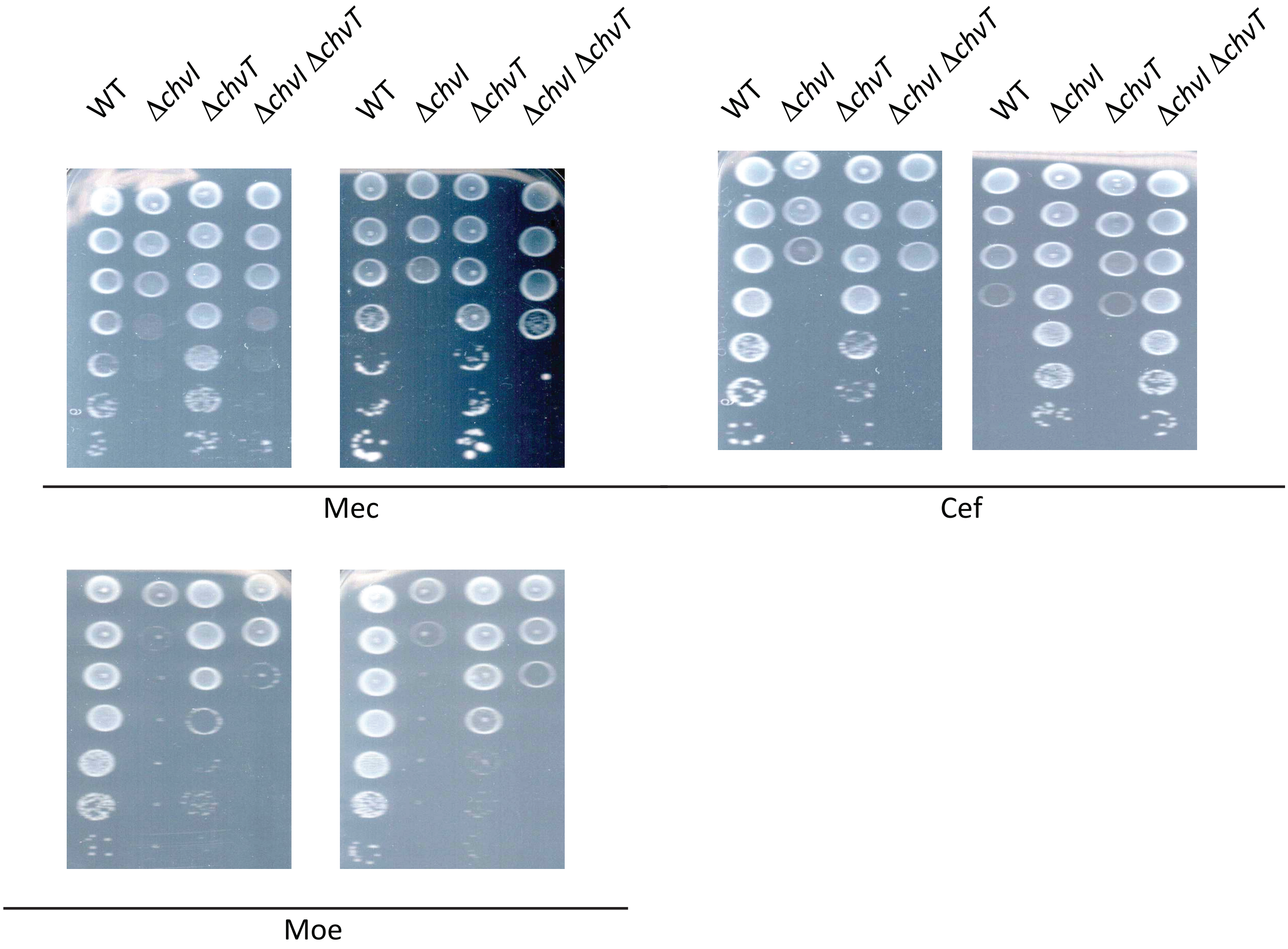
WT
ΔchvI
ΔchvT
ΔchvI ΔchvT
WT
ΔchvI
ΔchvT
ΔchvI ΔchvT
WT
ΔchvI
ΔchvT
ΔchvI ΔchvT
WT
ΔchvI
ΔchvT
ΔchvI ΔchvT
Mec
Cef
Moe
