# Supplementary material for: The two-component system ChvGI maintains cell envelope homeostasis in Caulobacter crescentus
Source: PLoS Genet. 2022 Dec 8;18(12):e1010465. doi: 10.1371/journal.pgen.1010465 (PMC9731502; doi:10.1371/journal.pgen.1010465)
Supplement: S2 Table — (A) genes with expression down-regulated or (B) up-regulated in the ΔchvI mutant upon osmotic stress with 6% sucrose. (PDF) [file pgen.1010465.s009.pdf]

S2 Table. ChvI regulon determined by RNA-seq. (A) genes with expression down-regulated in the Δ*chvI* mutant upon osmotic stress with 6% sucrose.

Top hit	Gene ID	Description	log2 (FC WT/Δ*chvI*)	P-value	P-adj
1	CCNA_03997	amelogenin/CpxP-related protein	8,579868	1,10E-239	4,43E-236
2	CCNA_00237	two-component response regulator chvI	7,460508	1,34E-191	2,72E-188
3	CCNA_R0088	Minimal medium sRNA	6,136769	1,77E-115	2,39E-112
4	CCNA_01238	EF-hand domain protein	5,332779	2,91E-107	2,95E-104
5	CCNA_R0092	Minimal medium sRNA	5,227363	8,42E-43	2,43E-40
6	CCNA_00889	hypothetical protein	4,883146	4,29E-86	3,47E-83
7	CCNA_R0161	small non-coding RNA	4,543405	2,92E-29	3,81E-27
8	CCNA_03987	hypothetical protein	4,275498	1,49E-46	5,03E-44
9	CCNA_01660	hypothetical protein	4,093043	3,23E-48	1,19E-45
10	CCNA_02817	retrotransposon-related protein	4,044014	7,82E-51	3,16E-48
11	CCNA_01087	cell envelope integrity protein eipA	4,041426	1,91E-53	9,67E-51
12	CCNA_03308	hypothetical protein	3,974113	5,99E-30	8,66E-28
13	CCNA_02309	EF hand domain protein	3,947459	6,60E-29	7,80E-27
14	CCNA_03102	hypothetical protein	3,784691	1,09E-33	1,77E-31
15	CCNA_03820	outer-membrane lipoproteins carrier protein	3,782962	8,49E-65	5,72E-62
16	CCNA_01080	hypothetical protein	3,690148	1,16E-40	2,92E-38
17	CCNA_02531	proline-rich hypothetical protein	3,486805	2,19E-26	2,01E-24
18	CCNA_03601	hemolysin III-like protein)	3,466022	2,92E-59	1,69E-56
19	CCNA_00038	hypothetical protein	3,459263	3,52E-42	9,49E-40
20	CCNA_01341	endopeptidase degP	3,299589	2,05E-52	9,23E-50
21	CCNA_03602	patatin family phospholipase domain protein	3,296568	2,80E-38	5,96E-36
22	CCNA_00687	hypothetical protein	3,203373	8,72E-39	2,08E-36
23	CCNA_00039	hypothetical protein	3,175555	1,20E-23	9,91E-22
24	CCNA_R0199	small non-coding RNA	3,138597	1,91E-35	3,36E-33
25	CCNA_01583	phosphate-binding protein PtsS	3,10072	7,81E-36	1,44E-33
26	CCNA_01592	SN-glycerol-3-phosphate transport ATP-binding protein	3,084726	4,35E-38	8,81E-36
27	CCNA_02165	esterase/lipase	3,081526	6,50E-22	4,96E-20
28	CCNA_01090	hypothetical protein	3,075921	1,72E-43	5,34E-41
29	CCNA_02310	cellulose 1,4-beta-cellobiosidase	3,060081	7,64E-30	1,07E-27
30	CCNA_02721	peptidase, M16 family	3,050148	9,87E-29	1,08E-26

31	CCNA_03101	integrase/recombinase (XerD/RipX family)	3,019754	3,27E-25	2,88E-23
32	CCNA_01067	type I secretion outer membrane protein RsaFa	2,98762	4,23E-27	4,17E-25
33	CCNA_01773	SNARE-associated family membrane protein	2,986556	5,91E-29	7,24E-27
34	CCNA_02106	TonB-dependent outer membrane receptor	2,967977	2,22E-28	2,30E-26
35	CCNA_00888	hypothetical protein	2,966904	3,27E-20	2,41E-18
36	CCNA_00686	peptidase family S41 protein	2,944474	1,82E-26	1,75E-24
37	CCNA_00484	hypothetical protein	2,926154	2,15E-26	2,01E-24
38	CCNA_02308	hypothetical protein	2,904541	3,41E-29	4,31E-27
39	CCNA_02846	endopeptidase degP	2,873802	6,85E-34	1,15E-31
40	CCNA_01088	hypothetical protein	2,86569	8,62E-23	6,98E-21
41	CCNA_02001	phosphoserine phosphatase	2,845159	6,07E-32	9,44E-30
42	CCNA_01445	hypothetical protein	2,833545	1,36E-38	3,05E-36
43	CCNA_01089	hypothetical protein	2,815112	9,13E-37	1,76E-34
44	CCNA_03726	transcriptional regulator	2,804138	2,20E-28	2,30E-26
45	CCNA_02219	hypothetical protein	2,733332	9,54E-26	8,58E-24
46	CCNA_03338	TolB protein	2,699714	3,38E-30	5,07E-28
47	CCNA_00733	GumN superfamily protein	2,600268	1,73E-29	2,33E-27
48	CCNA_01446	putative aminotransferase aatC	2,593795	2,07E-19	1,42E-17
49	CCNA_02339	methyltransferase	2,585514	3,45E-28	3,49E-26
50	CCNA_02889	peptidyl-prolyl cis-trans isomerase	2,585088	6,91E-29	7,80E-27
51	CCNA_00546	hypothetical protein	2,581795	1,17E-13	5,42E-12
52	CCNA_01505	hypothetical protein	2,578745	3,66E-11	1,42E-09
53	CCNA_01386	hypothetical protein	2,494249	2,47E-24	2,08E-22
54	CCNA_02865	phage protein	2,484474	3,01E-14	1,52E-12
55	CCNA_00735	hypothetical protein BamF	2,477619	8,96E-18	5,58E-16
56	CCNA_03909	conserved hypothetical protein	2,45932	6,94E-29	7,80E-27
57	CCNA_01653	peptidyl-prolyl cis-trans isomerase	2,417407	7,40E-16	3,99E-14
58	CCNA_00123	3-hydroxyacyl CoA dehydrogenase	2,40595	1,14E-13	5,38E-12
59	CCNA_03336	Tol system periplasmic component YbgF	2,320795	1,74E-24	1,50E-22
60	CCNA_02201	hypothetical protein	2,320121	6,67E-12	2,75E-10
61	CCNA_03548	carboxy-terminal processing protease precursor	2,277761	3,32E-16	1,84E-14
62	CCNA_R0100	small non-coding RNA chvR	2,245174	1,21E-08	3,46E-07
63	CCNA_01654	peptidyl-prolyl cis-trans isomerase	2,236579	3,61E-22	2,81E-20

64	CCNA_02819	hypothetical protein	2,214202	8,69E-12	3,52E-10
65	CCNA_R0106	small non-coding RNA	2,189526	5,66E-19	3,76E-17
66	CCNA_01081	hypothetical protein	2,177142	3,54E-21	2,65E-19
67	CCNA_01993	membrane endopeptidase MmpA	2,173333	3,37E-19	2,27E-17
68	CCNA_01725	PQQ enzyme repeat family protein BamA	2,166415	1,36E-19	9,66E-18
69	CCNA_01147	acetyltransferase	2,161138	5,71E-13	2,60E-11
70	CCNA_01772	hypothetical protein	2,159305	1,90E-16	1,07E-14
71	CCNA_00292	phosphate transport system permease protein pstC	2,148154	3,56E-22	2,81E-20
72	CCNA_01344	hypothetical protein	2,109334	7,12E-20	5,14E-18
73	CCNA_02202	hypothetical protein	2,085797	4,21E-14	2,08E-12
74	CCNA_02338	hypothetical protein	2,07658	3,46E-16	1,89E-14
75	CCNA_03212	hypothetical protein	2,024365	6,94E-12	2,83E-10
76	CCNA_01427	lipoprotein, SmpA/OmlA family BamE	2,013488	1,09E-06	2,17E-05
77	CCNA_03460	hypothetical protein	2,006656	3,40E-18	2,15E-16
78	CCNA_03335	PP-loop family cell cycle control ATPase	1,996091	9,45E-18	5,79E-16
79	CCNA_01443	hypothetical protein	1,974744	2,71E-17	1,59E-15
80	CCNA_01447	homoserine dehydrogenase	1,959219	2,88E-11	1,13E-09
81	CCNA_00793	phosphatidylglycerol glycosyltransferase	1,957657	3,58E-14	1,79E-12
82	CCNA_01500	hypothetical protein	1,953374	1,86E-18	1,22E-16
83	CCNA_03773	hypothetical protein	1,952802	1,74E-17	1,04E-15
84	CCNA_00218	hypothetical protein	1,94742	1,48E-19	1,03E-17
85	CCNA_00347	hypothetical protein	1,889342	0,000181 9	0,002055 5
86	CCNA_00887	hypothetical protein	1,873478	1,15E-12	5,01E-11
87	CCNA_01759	peptidyl-prolyl cis-trans isomerase	1,862241	1,02E-16	5,87E-15
88	CCNA_01659	queuine tRNA-ribosyltransferase	1,859312	1,46E-15	7,78E-14
89	CCNA_01724	hypothetical protein	1,854791	1,63E-16	9,30E-15
90	CCNA_01345	short chain dehydrogenase	1,825671	7,99E-13	3,55E-11
91	CCNA_01068	mannosyltransferase	1,82141	2,12E-15	1,10E-13
92	CCNA_03280	pyruvate ferredoxin/flavodoxin oxidoreductase family protein	1,821146	1,14E-17	6,88E-16
93	CCNA_00548	transposase	1,813886	0,000110 2	0,001308
94	CCNA_03083	hybrid sensor histidine kinase/receiver domain protein	1,792828	2,69E-13	1,23E-11
95	CCNA_01496	2-dehydro-3-deoxyphosphooctonate aldolase	1,787233	1,64E-14	8,40E-13

96	CCNA_00293	phosphate transport system permease protein pstA	1,75902	1,94E-15	1,02E-13
97	CCNA_01636	alkaline phosphatase	1,745158	1,15E-10	4,33E-09
98	CCNA_R0074	small non-coding RNA	1,742013	0,0006619	0,0063767
99	CCNA_03557	hypothetical protein	1,724285	2,54E-09	8,16E-08
100	CCNA_00398	hypothetical protein	1,721562	1,71E-12	7,28E-11
101	CCNA_02334	transcriptional regulator of stalk biogenesis staR	1,718166	5,69E-09	1,69E-07
102	CCNA_03469	arginyl-tRNA synthetase	1,690582	7,19E-13	3,23E-11
103	CCNA_03461	hypothetical protein	1,688066	3,43E-06	6,14E-05
104	CCNA_00124	hypothetical protein	1,685398	1,07E-13	5,10E-12
105	CCNA_03709	transposase	1,673242	3,44E-06	6,14E-05
106	CCNA_01385	predicted rRNA methylase	1,672307	2,05E-09	6,70E-08
107	CCNA_01907	ABC transporter ATP-binding protein	1,658489	1,58E-07	3,86E-06
108	CCNA_00707	hypothetical protein	1,650257	2,11E-10	7,67E-09
109	CCNA_02063	lipoprotein, ComL family BamD	1,649258	9,53E-14	4,59E-12
110	CCNA_02888	nitrilotriacetate monooxygenase	1,632957	2,29E-08	6,27E-07
111	CCNA_00238	two-component sensor histidine kinase chvG	1,626522	7,18E-14	3,50E-12
112	CCNA_02781	hypothetical protein	1,625061	3,84E-08	1,01E-06
113	CCNA_00169	hypothetical protein	1,611327	3,11E-12	1,31E-10
114	CCNA_R0121	small non-coding RNA	1,603412	2,25E-05	0,0003365
115	CCNA_03196	hypothetical protein	1,558725	3,57E-08	9,49E-07
116	CCNA_03341	TolQ protein	1,544807	3,49E-08	9,36E-07
117	CCNA_00272	RmuC family protein	1,53237	1,32E-12	5,69E-11
118	CCNA_03725	ATP-dependent DNA ligase	1,522201	1,73E-07	4,19E-06
119	CCNA_R0132	small non-coding RNA	1,51777	0,0001092	0,0013029
120	CCNA_00784	heat resistant agglutinin	1,514111	1,98E-05	0,0003046
121	CCNA_01992	outer membrane protein assembly factor BamB	1,510435	1,07E-12	4,73E-11
122	CCNA_03784	transporter	1,503962	3,22E-05	0,0004458
123	CCNA_01501	protein kinase C-like superfamily protein	1,497378	1,59E-05	0,0002484
124	CCNA_03117	cytosolic protein BacB	1,492925	1,93E-09	6,34E-08
125	CCNA_01994	1-deoxy-D-xylulose 5-phosphate reductoisomerase	1,491637	4,05E-06	7,06E-05
126	CCNA_00547	PurR-related transcriptional regulator	1,45568	4,07E-09	1,26E-07

127	CCNA_03506	transcriptional regulator, algH	1,45187	1,46E-11	5,81E-10
128	CCNA_02115	secreted pectate lyase-family protein	1,442696	1,29E-09	4,35E-08
129	CCNA_00907	3-oxoacyl-(acyl-carrier-protein) synthase III	1,440485	3,53E-10	1,23E-08
130	CCNA_01758	4-hydroxythreonine-4-phosphate dehydrogenase PdxA	1,436887	2,18E-10	7,89E-09
131	CCNA_00116	phosphoglucosamine mutase	1,435697	2,58E-10	9,17E-09
132	CCNA_00785	tRNA (5-aminomethyl-2-thiouridylate) methyltransferase/tRNA (5-carboxymethylaminomethyl-2-thiouridyl	1,418898	4,83E-05	0,000638 6
133	CCNA_02009	hypothetical protein	1,412129	8,88E-07	1,81E-05
134	CCNA_02838	EF hand domain protein	1,409921	5,98E-07	1,29E-05
135	CCNA_01497	ADP-L-glycero-D-manno-heptose-6-epimerase	1,408935	3,85E-09	1,20E-07
136	CCNA_03710	nitrogen regulatory EIIA_Ntr protein	1,407127	5,17E-09	1,57E-07
137	CCNA_01346	outer membrane protein	1,404111	2,24E-07	5,30E-06
138	CCNA_03772	HIT1 protein	1,399326	1,59E-08	4,47E-07
139	CCNA_03600	integral membrane protein	1,399002	8,07E-05	0,000989 4
140	CCNA_03172	3-oxoacyl-(acyl-carrier protein) reductase	1,396147	5,36E-07	1,17E-05
141	CCNA_00645	nitrate binding protein nrtA	1,378191	1,27E-05	0,000201 1
142	CCNA_00290	autotransporter protein	1,377947	2,48E-10	8,88E-09
143	CCNA_01607	hypothetical protein	1,370074	5,40E-09	1,63E-07
144	CCNA_01731	colicin V production protein	1,360457	4,81E-09	1,47E-07
145	CCNA_03211	L-Ala-D/L-Glu racemase	1,341752	3,98E-07	8,96E-06
146	CCNA_R0128	small non-coding RNA	1,341529	0,001147 9	0,010428 7
147	CCNA_02220	beta-glucosidase	1,336701	6,39E-07	1,36E-05
148	CCNA_00122	hypothetical protein	1,299073	5,63E-09	1,69E-07
149	CCNA_01019	beta-glucosidase	1,278072	7,01E-08	1,83E-06
150	CCNA_01613	rod shape-determining protein mreC	1,272769	3,96E-07	8,95E-06
151	CCNA_03599	aspartate-semialdehyde dehydrogenase	1,248873	0,000352 4	0,003675 1
152	CCNA_03340	TolR protein	1,245906	3,52E-06	6,25E-05
153	CCNA_02837	RNA polymerase ECF-type sigma factor	1,241975	7,74E-05	0,000952 7
154	CCNA_02820	hypothetical protein	1,226276	1,63E-06	3,13E-05
155	CCNA_00378	thiol:disulfide interchange protein dsbA	1,222306	2,00E-07	4,78E-06
156	CCNA_00882	hypothetical protein	1,20416	0,000264 8	0,002864 5

157	CCNA_03995	hypothetical protein	1,20192	0,000150 4	0,001728 2
158	CCNA_01708	hypothetical protein	1,191325	1,85E-07	4,46E-06
159	**CCNA_00130**	Kup system potassium uptake protein	1,19066	6,78E-05	0,000860 5
160	CCNA_00364	deoxyhypusine synthase	1,186074	4,65E-05	0,000621 4
161	**CCNA_02081**	Sec-independent protein translocase protein tatB	1,175577	3,07E-07	7,14E-06
162	CCNA_01020	transcriptional regulator, LacI family	1,173496	4,68E-05	0,000621 5
163	CCNA_01885	short chain dehydrogenase	1,17193	8,57E-05	0,001047 7
164	CCNA_03092	cytochrome P450 IVA5	1,166815	1,43E-07	3,52E-06
165	CCNA_00121	hypothetical protein	1,160166	9,85E-07	1,98E-05
166	**CCNA_02816**	hypothetical protein	1,154049	2,24E-05	0,000335 4
167	**CCNA_03711**	ribosome-associated factor Y	1,149614	3,29E-07	7,56E-06
168	CCNA_03882	phage gp6-like head-tail connector protein	1,14729	0,005454	0,039688 7
169	CCNA_01971	peptidyl-prolyl cis-trans isomerase	1,145348	1,49E-06	2,91E-05
170	CCNA_00096	L-asparaginase	1,1444	1,62E-06	3,13E-05
171	**CCNA_03339**	TolA protein	1,143314	6,38E-06	0,000107 1
172	CCNA_01569	transcriptional regulatory protein	1,141523	4,87E-05	0,000641 9
173	CCNA_00307	phospholipid-lipopolysaccharide ABC transporter	1,137902	7,09E-05	0,000893 6
174	CCNA_03318	cytosolic protein	1,136987	0,001303 2	0,011562 9
175	CCNA_R0048	tRNA Leu	1,128249	0,000112 6	0,001332 4
176	CCNA_03209	acetyl-coenzyme A synthetase	1,126587	0,001879 2	0,015873 5
177	CCNA_01503	hypothetical protein	1,122059	0,001096 2	0,010057 6
178	CCNA_00294	phosphate transport ATP-binding protein pstB	1,121301	5,37E-07	1,17E-05
179	CCNA_03782	heme exporter protein A	1,115029	0,000202 2	0,002247 9
180	**CCNA_01584**	multimodular transpeptidase-transglycosylase Pbp1a (PBP 1A)	1,112263	8,32E-07	1,70E-05
181	CCNA_01267	hypothetical protein	1,107908	5,96E-05	0,000769 9
182	**CCNA_01268**	cytochrome c	1,102669	2,23E-07	5,30E-06
183	**CCNA_00486**	TonB-dependent receptor	1,101377	4,21E-05	0,000565 5
184	CCNA_03783	heme exporter protein B	1,100177	0,000423 4	0,004336 9
185	CCNA_02129	acyl-CoA dehydrogenase, short-chain specific	1,099674	1,40E-06	2,75E-05

186	CCNA_03487	hypothetical protein	1,091469	1,11E-05	0,000180<br>2
187	CCNA_03357	hypothetical protein	1,087396	0,001023<br>6	0,009477<br>5
188	CCNA_03175	cytosolic protein	1,083812	0,000113<br>4	0,001338
189	CCNA_03786	heme chaperone heme-lyase	1,083588	1,96E-06	3,70E-05
190	CCNA_R0180	small non-coding RNA	1,08319	5,50E-05	0,000715<br>9
191	CCNA_00359	SH3 domain-containing cell surface protein	1,079742	0,000304<br>5	0,003241<br>8
192	CCNA_02653	sensory transduction protein kinase	1,079419	9,21E-05	0,001116
193	CCNA_03864	cytosolic protein	1,077941	8,31E-06	0,000137<br>7
194	CCNA_03621	transcriptional regulator, AraC family	1,076176	2,29E-05	0,000339<br>9
195	CCNA_01991	hypothetical protein	1,074656	1,90E-06	3,61E-05
196	CCNA_02064	UDP-3-O-(3-hydroxymyristoyl) N-acetylglucosamine deacetylase LpxC	1,069027	2,84E-06	5,18E-05
197	CCNA_03020	hypothetical protein	1,054913	2,58E-06	4,81E-05
198	CCNA_01663	potassium-transporting ATPase A chain	1,03838	0,001118<br>6	0,010193
199	CCNA_01732	DNA repair protein RadA	1,024789	2,41E-05	0,000351<br>7
200	CCNA_02075	peptidoglycan-specific endopeptidase, M23 family DipM	1,016395	2,94E-05	0,000417<br>2
201	CCNA_01831	tryptophan halogenase superfamily protein	1,011794	0,004722<br>8	0,034869<br>3
202	CCNA_00978	transposase	1,011037	0,007065<br>8	0,048537<br>2
203	CCNA_02082	Sec-independent protein translocase protein tatA	0,996894	0,000225<br>2	0,002482<br>3
204	CCNA_01082	hypothetical protein	0,991064	0,001114<br>3	0,010177<br>4
205	CCNA_01709	hypothetical protein	0,985944	7,12E-05	0,000893<br>9
206	CCNA_01442	N-acetyl-gamma-glutamyl-phosphate reductase	0,982854	0,000385<br>1	0,003965<br>1
207	CCNA_00379	thiol:disulfide interchange protein dsbA	0,980262	0,000102<br>4	0,001232<br>5
208	CCNA_02794	asparagine synthetase (glutamine-hydrolyzing)	0,979714	0,000273<br>1	0,002946<br>1
209	CCNA_03671	predicted phosphohydrolase, Icc family	0,977623	0,002628	0,021351<br>3
210	CCNA_01095	phenylalanyl-tRNA synthetase subunit beta	0,971444	0,002215<br>3	0,018329<br>2
211	CCNA_03791	transcriptional regulator, MarR family	0,965848	4,00E-05	0,000539<br>4
212	CCNA_03091	hypothetical protein NstA	0,965182	0,000296<br>4	0,003172<br>8
213	CCNA_01955	zinc metalloprotease	0,963472	0,000197<br>7	0,002216<br>2

214	**CCNA_01638**	beta-lactamase family protein	0,96309	6,39E-05	0,0008186
215	**CCNA_03356**	cytosolic protein "zapA binds to FtsZ"	0,958486	0,0007594	0,0072468
216	CCNA_00170	TonB-dependent receptor	0,956425	5,86E-05	0,0007605
217	CCNA_00196	3-isopropylmalate dehydratase large subunit	0,954461	0,0004716	0,0047585
218	CCNA_02378	hypothetical protein	0,953957	0,0032419	0,0254201
219	**CCNA_01612**	rod shape-determining protein mreB	0,948152	2,52E-05	0,0003646
220	CCNA_00138	TonB-dependent receptor	0,946772	2,62E-05	0,0003774
221	CCNA_02955	putative permease	0,943332	0,0016803	0,0143432
222	CCNA_03459	methyl-accepting chemotaxis protein	0,940613	7,14E-05	0,0008939
223	CCNA_01342	ATPase, AAA family	0,939596	2,35E-05	0,0003472
224	CCNA_01504	ATP-dependent DNA helicase recG	0,922947	2,39E-05	0,0003515
225	CCNA_00906	hypothetical protein	0,922502	0,0001983	0,0022164
226	**CCNA_02008**	prolyl-tRNA synthetase	0,916529	2,48E-05	0,0003604
227	CCNA_02942	hypothetical protein	0,907706	5,40E-05	0,0007043
228	CCNA_00120	acetyltransferase	0,902977	0,0005857	0,0057935
229	**CCNA_03838**	gluconate 2-dehydrogenase/glyoxylate reductase/hydroxypyruvate reductase	0,902758	0,0031848	0,0250206
230	CCNA_03780	hypothetical protein	0,8922	0,0006172	0,0060171
231	CCNA_01028	cytosol aminopeptidase	0,88544	0,0052442	0,0383692
232	**CCNA_03716**	hypothetical protein	0,884475	0,000217	0,0023983
233	CCNA_01448	fructose-1,6-bisphosphatase	0,884159	0,0003022	0,0032265
234	CCNA_02325	ornithine carbamoyltransferase	0,881094	0,0021261	0,0176278
235	CCNA_03488	hypothetical protein	0,879008	0,0005483	0,0054644
236	CCNA_01306	LSU ribosomal protein L3P	0,874311	0,002926	0,0234437
237	CCNA_01582	hypothetical protein	0,872429	0,001489	0,0129842
238	CCNA_00295	phosphate transport system protein phoU	0,865636	0,0001425	0,0016522
239	CCNA_02317	hypothetical protein	0,860708	0,0011712	0,0105901
240	CCNA_01664	potassium-transporting ATPase B chain	0,858797	0,0070552	0,0485372

241	CCNA_03305	SSU ribosomal protein S7P	0,855205	0,0001433	0,0016564
242	CCNA_02326	acetylornithine aminotransferase/succinyldiaminopimelate aminotransferase	0,853277	0,0018229	0,0154299
243	**CCNA_03837**	hypothetical protein	0,847624	0,0012026	0,0107415
244	CCNA_01416	3-hydroxyisobutyrate dehydrogenase	0,8471	0,0071303	0,048732
245	CCNA_R0162	small non-coding RNA	0,838332	0,0040137	0,0305251
246	CCNA_01891	enoyl-CoA hydratase/carnithine racemase	0,835318	0,0016618	0,0142355
247	**CCNA_00419**	fasciclin domain cell surface protein	0,834796	0,0001816	0,0020555
248	CCNA_01026	hypothetical protein	0,828752	0,0025994	0,0212043
249	CCNA_03174	permease	0,827187	0,0011038	0,0101041
250	**CCNA_00517**	peptidyl-tRNA hydrolase	0,82139	0,0035583	0,0274224
251	**CCNA_01614**	hypothetical protein mreD	0,800707	0,0026613	0,0215783
252	CCNA_00420	hypothetical protein	0,800279	0,0015977	0,0138425
253	CCNA_03304	protein translation elongation factor G (EF-G)	0,791329	0,0044151	0,0332037
254	CCNA_00360	putative hydrolase	0,791311	0,006858	0,0475943
255	CCNA_01980	methylisocitrate lyase	0,788602	0,0005956	0,0058778
256	CCNA_00151	ribonuclease PH	0,786424	0,000537	0,0053743
257	**CCNA_03727**	small-conductance mechanosensitive channel mcsS	0,785585	0,0004668	0,0047344
258	CCNA_00085	dienelactone hydrolase-related protein	0,775303	0,0019485	0,0163564
259	CCNA_02480	benzaldehyde dehydrogenase (NAD+)	0,771528	0,0067963	0,0473065
260	CCNA_03351	phosphoribosylamidoimidazole-succinocarboxamide synthase	0,768125	0,0044534	0,0333556
261	**CCNA_00519**	hypothetical protein	0,766235	0,0007975	0,0075746
262	CCNA_01863	outer membrane efflux protein	0,76103	0,0066595	0,0466973
263	**CCNA_01438**	heme O monooxygenase	0,757038	0,0013238	0,0116947
264	CCNA_00794	hypothetical protein	0,756107	0,0036754	0,0281111
265	CCNA_01330	DNA-directed RNA polymerase subunit alpha	0,751066	0,0021012	0,0175287
266	**CCNA_00377**	chromosome partition protein smc	0,745145	0,0009538	0,0089334
267	CCNA_02923	TonB-dependent receptor	0,740021	0,0041079	0,0311827

268	CCNA_03865	leucyl-tRNA synthetase	0,739799	0,0017797	0,0151272
269	CCNA_02370	TonB-dependent maltose outer membrane transporter malA	0,725134	0,0015128	0,0131629
270	CCNA_01252	soluble lytic murein transglycosylase SdpA	0,72267	0,0026258	0,0213513
271	CCNA_03307	hypothetical protein	0,721804	0,0009621	0,0089902
272	CCNA_R0039	tRNA Arg	0,719034	0,0034881	0,0270358
273	**CCNA_03195**	RNA polymerase sigma-32 factor	0,715546	0,0044321	0,0332696
274	CCNA_03735	transketolase	0,712932	0,003036	0,0240387
275	CCNA_00306	hypothetical protein	0,710158	0,0069937	0,0482053
276	CCNA_01546	DEAD-box RNA helicase-like protein	0,709828	0,0058513	0,042125
277	CCNA_01793	hypothetical protein	0,705985	0,0011785	0,0106196
278	CCNA_00839	glucan 1,4-beta-glucosidase	0,702798	0,007212	0,0492068
279	CCNA_03752	UTP-glucose-1-phosphate uridylyltransferase	0,697334	0,0062747	0,0447749
280	**CCNA_00585**	hypothetical protein	0,696581	0,002363	0,0194722
281	CCNA_00037	esterase lipase family protein	0,696386	0,0029813	0,0236985
282	CCNA_02583	phosphoribosylformylglycinamidine synthase I	0,690972	0,0030918	0,0244326
283	**CCNA_02007**	lipoprotein releasing system transmembrane protein lolE	0,685244	0,0017197	0,0146479
284	CCNA_00778	GTP-binding protein TypA/BipA	0,683274	0,0062358	0,0445761
285	CCNA_01637	topoisomerase IV subunit A	0,672635	0,0025727	0,0210285
286	**CCNA_03682**	fumarylpyruvate hydrolase	0,669784	0,0018119	0,015369
287	CCNA_01956	outer membrane protein	0,66209	0,0029497	0,0235863
288	CCNA_02232	TonB-dependent receptor	0,659847	0,0059121	0,042487
289	**CCNA_03683**	maleylpyruvate isomerase	0,658792	0,0044601	0,0333556
290	CCNA_00495	cytosolic protein	0,658202	0,0034508	0,0267984
291	CCNA_01990	UDP-3-O-(3-hydroxymyristoyl) glucosamine N-acyltransferase	0,656367	0,0067828	0,0473065
292	**CCNA_01060**	type I protein secretion ATP-binding protein RsaD	0,638711	0,003365	0,0261824
293	CCNA_03879	uroporphyrinogen decarboxylase	0,63689	0,0041681	0,0315807
294	CCNA_01851	quinol cytochrome oxidase polypeptide II	0,627876	0,0064339	0,0455141

295	CCNA_02406	membrane lipoprotein	0,624075	0,005955 8	0,042725 8
296	CCNA_00483	Zn-dependent hydrolase, glyoxalase II family	0,620069	0,006674 6	0,046722 4
297	CCNA_03306	SSU ribosomal protein S12P	0,614043	0,004495 9	0,033499 9
298	CCNA_01237	hypothetical protein	0,609086	0,006804 8	0,047306 5
299	CCNA_01650	copper/zinc superoxide dismutase	0,605626	0,005622 2	0,040693
300	CCNA_01223	acyl-CoA synthetase	0,600935	0,006732 5	0,047045 8
301	CCNA_00029	lysine exporter protein	0,596043	0,007098 8	0,048598 6

Genes highlighted in green were identified in the ChIP-seq regulon in this work, and those in yellow the RNA-seq regulon presented by [1].

(B) genes with expression up-regulated in the Δ*chvI* mutant upon osmotic stress with 6% sucrose.

Top hit	Gene ID	Description	log2 (FC WT/Δ*chvI*)	P-value	P-adj
1	CCNA_01202	membrane alanine aminopeptidase	-1,933379	2,36E-18	1,51E-16
2	**CCNA_00438**	hypothetical protein	-1,913719	5,69E-06	9,67E-05
3	**CCNA_01303**	hypothetical protein	-1,882275	1,16E-11	4,64E-10
4	**CCNA_00974**	OAR protein precursor	-1,789948	1,36E-08	3,85E-07
5	CCNA_R0061	RNase P RNA	-1,754613	5,70E-07	1,24E-05
6	CCNA_01523	acetyltransferase flmH	-1,690376	1,57E-10	5,81E-09
7	**CCNA_03108**	ChvT TonB-dependent outer membrane receptor	-1,689341	8,08E-07	1,66E-05
8	CCNA_01121	hypothetical protein	-1,678219	4,09E-11	1,56E-09
9	**CCNA_02200**	cytochrome c-family protein	-1,647808	8,55E-08	2,19E-06
10	**CCNA_00444**	chemotaxis protein methyltransferase	-1,642055	1,23E-10	4,60E-09
11	CCNA_01186	hypothetical protein	-1,62859	0,00026	0,0028554
12	CCNA_03043	type IV pilin protein pilA	-1,601108	0,0001	0,0012221
13	CCNA_03395	two-component receiver domain protein	-1,579211	1,27E-05	0,0002011
14	**CCNA_03325**	hypothetical protein	-1,550968	7,06E-07	1,47E-05
15	CCNA_01476	CRP-family transcription regulator ftrB	-1,530608	2,61E-10	9,17E-09
16	**CCNA_01269**	GcrB protein	-1,52671	2,10E-09	6,78E-08
17	CCNA_02515	cytosolic protein	-1,524018	6,02E-07	1,30E-05
18	**CCNA_00417**	hypothetical protein	-1,522626	3,87E-09	1,20E-07
19	CCNA_00948	hypothetical protein =SciP	-1,515411	3,61E-12	1,51E-10
20	CCNA_03607	ribonucleoside-diphosphate reductase subunit alpha	-1,505469	3,53E-09	1,11E-07

21	CCNA_00709	hypothetical protein	-1,500115	2,67E-05	0,0003829
22	**CCNA_00446**	chemotaxis receiver domain protein cheYII	-1,493908	1,22E-09	4,15E-08
23	CCNA_03896	conserved hypothetical protein	-1,491758	1,39E-09	4,63E-08
24	CCNA_03136	flagellar export protein FliJ	-1,489151	3,63E-07	8,31E-06
25	CCNA_01475	OmpW family outer membrane protein	-1,484898	2,35E-08	6,38E-07
26	CCNA_01901	lipoprotein	-1,464359	1,97E-10	7,26E-09
27	**CCNA_00439**	methyl-accepting chemotaxis protein McpA	-1,453836	3,78E-08	9,99E-07
28	CCNA_02931	flgE-related flagellar hook protein	-1,447194	4,01E-06	7,03E-05
29	CCNA_03700	large-conductance mechanosensitive channel	-1,445665	1,59E-06	3,09E-05
30	**CCNA_03313**	hypothetical protein	-1,445108	3,80E-06	6,71E-05
31	CCNA_03944	hypothetical protein	-1,441941	0,00032	0,0033646
32	CCNA_01270	hypothetical protein	-1,438841	1,91E-08	5,30E-07
33	CCNA_03355	acylamino-acid-releasing enzyme	-1,43795	3,74E-11	1,44E-09
34	CCNA_00350	hypothetical protein	-1,435351	3,40E-06	6,14E-05
35	**CCNA_00440**	virus protein	-1,423983	3,91E-07	8,90E-06
36	CCNA_02142	flagellar basal-body rod protein flgF	-1,420865	3,23E-07	7,47E-06
37	CCNA_01526	flagellar biosynthesis regulatory protein flaF	-1,414115	2,00E-05	0,0003071
38	CCNA_01201	hypothetical protein	-1,412886	1,01E-08	2,90E-07
39	CCNA_02604	host cell attachment protein	-1,411122	2,66E-06	4,94E-05
40	CCNA_02257	fliN family protein	-1,390661	0,0003	0,003169
41	**CCNA_00441**	chemotaxis receiver domain protein cheYI	-1,368874	2,95E-09	9,41E-08
42	**CCNA_00628**	chemotaxis protein cheY	-1,36271	1,37E-07	3,37E-06
43	CCNA_01278	histidine phosphotransferase domain protein	-1,361569	0,00013	0,0014602
44	CCNA_R0176	small non-coding RNA	-1,360767	0,00618	0,044279
45	CCNA_01926	two-component response regulator DgcB	-1,351856	1,43E-09	4,74E-08
46	CCNA_02754	parathion hydrolase	-1,350412	7,05E-10	2,44E-08
47	CCNA_00835	flagellin	-1,348627	4,28E-07	9,56E-06
48	CCNA_01157	protoporphyrinogen oxidase	-1,348367	7,48E-10	2,56E-08
49	CCNA_01528	flagellin fljK	-1,345288	1,07E-06	2,14E-05
50	CCNA_00447	chemotaxis protein cheD	-1,33167	2,67E-07	6,27E-06
51	CCNA_02712	holdfast attachment protein hfaB	-1,323383	3,98E-06	7,00E-05
52	**CCNA_00166**	HvyA	-1,318296	8,98E-08	2,28E-06

53	CCNA_03246	hypothetical protein	-1,312915	1,11E-06	2,19E-05
54	CCNA_00943	flagellar hook-associated protein FlaN	-1,312769	6,38E-09	1,88E-07
55	**CCNA_00236**	hypothetical protein	-1,307781	7,38E-06	0,0001229
56	CCNA_00942	flagellar hook-associated protein FlgL	-1,304491	8,56E-08	2,19E-06
57	CCNA_00953	flagellar motor switch protein FliN	-1,304384	8,33E-08	2,16E-06
58	CCNA_00437	methyl-accepting chemotaxis protein	-1,299406	7,59E-07	1,57E-05
59	CCNA_00443	chemotaxis protein cheW	-1,295113	2,09E-05	0,0003179
60	CCNA_02667	flagellar basal-body protein flbY	-1,291132	6,53E-07	1,38E-05
61	CCNA_02711	holdfast attachment protein hfaA	-1,288624	1,25E-05	0,0001986
62	**CCNA_00442**	chemotaxis histidine kinase protein cheAI	-1,282375	1,67E-08	4,65E-07
63	CCNA_02922	hypothetical protein	-1,274303	0,00013	0,0015455
64	CCNA_00348	probable chemoreceptor Y4FA	-1,27202	6,79E-09	1,99E-07
65	CCNA_02845	two-component response regulator	-1,26644	2,01E-08	5,53E-07
66	CCNA_02199	methyltransferase	-1,263917	2,97E-07	6,95E-06
67	CCNA_02143	flagellar basal-body rod protein flgG	-1,263858	1,06E-07	2,67E-06
68	**CCNA_01034**	TonB-dependent outer membrane receptor	-1,258003	7,64E-09	2,22E-07
69	CCNA_00542	hypothetical protein	-1,257973	0,00039	0,0040559
70	CCNA_00382	adenine-specific methyltransferase ccrM	-1,250604	8,30E-09	2,40E-07
71	CCNA_02547	response regulator receiver protein divK	-1,246185	2,98E-05	0,0004207
72	CCNA_03999	hypothetical protein	-1,241266	2,38E-06	4,45E-05
73	CCNA_03539	hypothetical protein	-1,23881	1,32E-07	3,28E-06
74	CCNA_01477	oxygen-independent coproporphyrinogen-III oxidase hemN	-1,2367	1,08E-07	2,71E-06
75	CCNA_01365	aspartyl protease perP	-1,235193	2,98E-05	0,0004207
76	CCNA_00538	methyl-accepting chemotaxis protein	-1,234609	3,06E-08	8,26E-07
77	CCNA_00426	very-short-patch-repair endonuclease	-1,233175	0,00062	0,0060171
78	CCNA_02666	chemotactic signal-response protein cheL	-1,208915	1,21E-05	0,0001936
79	CCNA_01529	hypothetical protein	-1,204067	1,14E-05	0,0001833
80	CCNA_00803	chemotaxis protein cheW	-1,189911	6,28E-07	1,34E-05
81	CCNA_00965	chaperone protein DnaJ	-1,188986	3,07E-05	0,0004289
82	CCNA_00354	hypothetical protein	-1,185738	0,00043	0,0044134
83	CCNA_02105	hypothetical protein	-1,181311	0,00095	0,0089075
84	CCNA_02145	flagellar L-ring protein flgH	-1,181267	9,66E-07	1,95E-05
85	CCNA_02844	antitoxin protein parD-3	-1,180372	2,78E-06	5,08E-05

86	CCNA_00027	2OG-Fe(II) oxygenase	-1,175011	1,01E-05	0,0001645
87	CCNA_02665	flagellar P-ring protein flgI	-1,165739	1,60E-06	3,10E-05
88	CCNA_02363	hypothetical protein	-1,160395	0,00019	0,0021641
89	CCNA_01644	chemotaxis motB protein	-1,158135	6,49E-07	1,37E-05
90	CCNA_02141	flagellar fliL protein	-1,157392	2,22E-05	0,0003339
91	CCNA_00790	hypoxia negative feedback regulator FixT	-1,157196	0,00047	0,0047344
92	CCNA_02408	hypothetical protein	-1,155134	0,00032	0,0034072
93	CCNA_03932	hypothetical protein	-1,155059	5,03E-07	1,11E-05
94	CCNA_03993	hypothetical protein	-1,15475	0,00389	0,0296058
95	CCNA_00081	hypothetical protein	-1,153832	2,19E-05	0,0003313
96	CCNA_01032	RNA polymerase ECF-type sigma factor	-1,151891	0,00032	0,003351
97	CCNA_01119	hypothetical protein	-1,150537	0,00033	0,0034473
98	CCNA_02513	holdfast synthesis gene hfsA, frameshifted variant	-1,145246	0,00024	0,002637
99	CCNA_03762	hypothetical protein	-1,14113	0,0006	0,0059093
100	**CCNA_00471**	GDP-L-fucose synthase	-1,140668	3,43E-06	6,14E-05
101	CCNA_00094	probable UDP-N-acetyl-D-mannosaminuronic acid transferase	-1,138585	6,60E-07	1,38E-05
102	CCNA_03691	KidO	-1,137173	6,14E-05	0,0007892
103	CCNA_00729	hypothetical protein	-1,135676	1,83E-05	0,0002844
104	**CCNA_00445**	receiver domain-glutamate methylesterase cheBI	-1,1356	1,27E-07	3,17E-06
105	CCNA_03923	hypothetical protein	-1,134216	0,00059	0,0057935
106	CCNA_02139	polar flagellum positioning protein pflI	-1,131809	5,59E-06	9,56E-05
107	CCNA_R0117	small non-coding RNA	-1,127058	3,04E-05	0,0004263
108	**CCNA_00472**	GDP-mannose 4,6 dehydratase	-1,123613	4,86E-07	1,08E-05
109	**CCNA_00416**	hypothetical protein	-1,12286	2,69E-06	4,96E-05
110	**CCNA_00448**	cheU protein	-1,119834	4,31E-06	7,42E-05
111	CCNA_00946	Basal-body rod modification protein FlgD	-1,115472	1,80E-06	3,44E-05
112	CCNA_00093	hypothetical protein	-1,113679	3,14E-05	0,0004364
113	CCNA_02196	hypothetical protein	-1,110725	7,34E-05	0,0009168
114	CCNA_00065	hypothetical protein	-1,108239	0,00012	0,0013551
115	CCNA_03037	pilus assembly ATPase CpaF	-1,106773	3,23E-05	0,0004458
116	CCNA_02534	hypothetical protein	-1,10674	0,00298	0,0236972
117	CCNA_02364	methyl-accepting chemotaxis protein	-1,102917	7,75E-07	1,60E-05
118	CCNA_03120	chemotaxis protein cheW	-1,099605	2,02E-05	0,000308

119	CCNA_01524	FlbA protein	-1,099099	4,29E-06	7,42E-05
120	CCNA_01532	regulatory protein flaY	-1,097971	1,56E-05	0,0002447
121	**CCNA_01685**	hemimethylated DNA-binding protein yccV	-1,082185	0,00093	0,0088028
122	**CCNA_00787**	chemotaxis motA protein	-1,078746	4,68E-05	0,0006215
123	CCNA_02546	GGDEF/response regulator protein pleD	-1,073107	0,00071	0,0068204
124	CCNA_03915	hypothetical protein	-1,071251	0,00236	0,0194722
125	CCNA_02722	hypothetical protein	-1,068107	2,29E-05	0,0003399
126	CCNA_02597	hypothetical protein	-1,067281	8,60E-06	0,000142
127	CCNA_02621	CAAX amino terminal protease family	-1,063135	9,89E-06	0,0001621
128	CCNA_01108	nucleoside-diphosphate-sugar epimerase	-1,058775	6,17E-06	0,0001041
129	CCNA_03039	pilus assembly protein CpaD	-1,058212	3,45E-05	0,0004734
130	CCNA_02720	hypothetical protein	-1,057207	0,00036	0,0037039
131	CCNA_02517	hypothetical protein	-1,056371	4,21E-06	7,31E-05
132	**CCNA_00629**	methyl-accepting chemotaxis protein	-1,054025	2,12E-05	0,0003215
133	CCNA_02622	M61 glycyl aminopeptidase	-1,0535	2,70E-06	4,96E-05
134	CCNA_01424	hypothetical protein	-1,052211	3,34E-06	6,06E-05
135	CCNA_01163	ice nucleation protein	-1,048322	5,60E-06	9,56E-05
136	CCNA_03042	pilus assembly prepilin peptidase CpaA	-1,03898	0,00063	0,00607
137	CCNA_02976	hypothetical protein	-1,037237	6,54E-05	0,0008344
138	CCNA_02540	N-acyl-L-amino acid amidohydrolase	-1,036741	1,18E-06	2,33E-05
139	CCNA_03763	deacetylase	-1,036727	7,12E-06	0,000119
140	CCNA_00834	flagellin	-1,034313	5,22E-05	0,0006838
141	CCNA_02514	polysaccharide secretin protein hfsD	-1,031577	0,00095	0,0089075
142	CCNA_03265	CBS pair-family sensor histidine kinase/receiver domain protein	-1,028312	0,00034	0,0035669
143	**CCNA_00982**	transcriptional regulator	-1,024448	0,00121	0,0107782
144	CCNA_01466	hypothetical protein	-1,024376	0,00693	0,0478721
145	CCNA_03295	PAS-family sensor histidine kinase	-1,021901	2,75E-05	0,0003915
146	CCNA_00950	flagellar M-ring protein FliF	-1,020532	2,18E-06	4,09E-05
147	CCNA_03247	methyl-accepting chemotaxis protein	-1,0199	1,12E-05	0,0001802
148	CCNA_00592	hypothetical protein	-1,016761	0,00053	0,005313
149	CCNA_03940	hypothetical protein	-1,014706	0,0029	0,0233382
150	CCNA_02680	hypothetical protein	-1,014085	5,82E-06	9,86E-05
151	CCNA_03062	cell wall hydrolase family protein	-1,006635	1,80E-05	0,0002808

152	CCNA_01952	N-acetylmuramoyl-L-alanine amidase AmiC	-1,004564	0,00043	0,0044134
153	CCNA_01258	permease	-1,000021	0,00025	0,002733
154	CCNA_02831	hypothetical protein	-0,999066	2,40E-05	0,0003515
155	CCNA_02713	holdfast attachment protein hfaD	-0,998279	7,75E-05	0,0009527
156	CCNA_03660	Usg protein	-0,995798	0,00484	0,0356356
157	CCNA_03202	hypothetical protein	-0,993089	0,00022	0,0023983
158	CCNA_02359	hypothetical protein	-0,991779	6,14E-05	0,0007892
159	CCNA_R0188	small non-coding RNA	-0,981879	0,00643	0,0455141
160	CCNA_00454	transcriptional regulator, GntR family	-0,981577	3,24E-05	0,0004458
161	CCNA_03585	chemotaxis receiver domain protein cheYIV	-0,98132	0,00099	0,0092568
162	CCNA_00951	flagellar motor switch protein FliG	-0,978554	2,49E-05	0,0003609
163	**CCNA_00224**	hypothetical protein	-0,976234	4,58E-05	0,0006129
164	CCNA_01122	hypothetical protein	-0,975603	0,00066	0,0063429
165	CCNA_03036	TadB-related pilus assembly protein	-0,975251	0,00117	0,0105901
166	CCNA_03198	two-component response regulator	-0,973815	0,00166	0,0142355
167	CCNA_02411	putative lytic transglycosylase pleA	-0,971665	0,00115	0,0104287
168	CCNA_03414	NAD(P) transhydrogenase alpha subunit	-0,969938	8,84E-06	0,0001453
169	CCNA_00945	chemotaxis protein MotD	-0,967557	0,0001	0,0012326
170	**CCNA_00302**	parathion hydrolase	-0,967207	1,03E-05	0,0001675
171	CCNA_03287	transcriptional regulatory protein	-0,95314	0,00036	0,0037595
172	CCNA_02625	cell division protein ftsQ	-0,953117	0,00011	0,0013044
173	CCNA_03839	acylamino-acid-releasing enzyme	-0,951083	0,00012	0,0014597
174	CCNA_01779	hypothetical protein	-0,948325	0,0006	0,0059169
175	CCNA_01257	permease	-0,948023	0,00058	0,0057709
176	CCNA_03424	AAA-family response regulator tacA	-0,947344	1,89E-05	0,0002916
177	CCNA_03249	TonB-dependent receptor	-0,946453	0,00024	0,0026626
178	CCNA_02643	division specific D,D-transpeptidase/cell division protein ftsI	-0,945101	9,09E-05	0,0011042
179	CCNA_00854	metallo-beta-lactamase protein	-0,944003	1,44E-05	0,000227
180	**CCNA_01035**	gamma-glutamyltranspeptidase	-0,936811	3,91E-05	0,0005293
181	CCNA_03137	endo-1,4-beta-xylanase	-0,936141	0,00064	0,0062338
182	CCNA_01525	flagellar biosynthesis repressor flbT	-0,932605	0,0012	0,0107399
183	CCNA_03135	flagellum-specific ATP synthase fliL	-0,932485	4,98E-05	0,0006548
184	CCNA_01004	flagellar basal-body rod protein FlgB	-0,932255	0,00647	0,045581

185	CCNA_02949	hypothetical protein	-0,930419	0,00011	0,0012951
186	CCNA_01185	hypothetical protein	-0,928673	3,47E-05	0,0004736
187	CCNA_03089	hypothetical protein	-0,927527	3,75E-05	0,0005094
188	CCNA_02238	hypothetical protein	-0,925002	0,00565	0,0407857
189	CCNA_02360	beta-D-Glcp beta-1,4-glucosyltransferase	-0,924491	0,00032	0,0033908
190	CCNA_01465	methyl-accepting chemotaxis protein	-0,921667	6,90E-05	0,000873
191	CCNA_02644	putative cell division protein	-0,919761	0,00293	0,0234437
192	CCNA_00627	hypothetical protein	-0,919012	0,00439	0,0330854
193	CCNA_03169	hypothetical protein	-0,917501	0,00028	0,0029801
194	CCNA_00234	WecE-family cell wall biogenesis enzyme	-0,916928	9,08E-05	0,0011042
195	CCNA_03686	alpha2 macroglobulin domain-containing extracellular protein	-0,916167	3,02E-05	0,0004237
196	CCNA_03130	cell cycle response regulator ctrA	-0,915187	0,00101	0,0093773
197	CCNA_03412	NAD(P) transhydrogenase subunit beta	-0,913158	2,74E-05	0,0003915
198	CCNA_03410	peptidoglycan-specific endopeptidase, M23 family LdpE	-0,902956	0,00146	0,0127708
199	CCNA_02719	hypothetical protein	-0,901484	0,00161	0,0138884
200	CCNA_03754	glutathione S-transferase family protein FzlA	-0,89973	0,00133	0,0117216
201	CCNA_01006	flagellar hook-basal body complex protein FliE	-0,897561	0,00208	0,0173953
202	CCNA_03413	NAD(P) transhydrogenase alpha subunit	-0,89729	6,65E-05	0,0008463
203	CCNA_02642	UDP-N-acetylmuramoylalanyl-D-glutamate--2, 6-diaminopimelate ligase	-0,896947	7,52E-05	0,0009332
204	CCNA_00875	Flp/Fap pilin component protein	-0,896139	0,00367	0,0280942
205	CCNA_00967	transcriptional regulator, TetR family	-0,895939	0,00016	0,0018596
206	CCNA_01248	transcriptional regulator, TetR family	-0,894381	7,66E-05	0,0009472
207	CCNA_00233	UDP-N-acetylglucosamine 4,6-dehydratase FlaA1	-0,892404	3,69E-05	0,000503
208	CCNA_03327	hypothetical protein	-0,889859	0,00447	0,0333915
209	CCNA_02950	hypothetical protein	-0,889715	0,00038	0,0039048
210	CCNA_03326	two-component sensor histidine kinase	-0,883349	0,00364	0,0279109
211	CCNA_00089	MHYT/PAS-family GGDEF/EAL protein	-0,869832	0,00017	0,0019556
212	CCNA_00591	catalase	-0,869626	0,00034	0,0035669
213	CCNA_02910	TonB-dependent receptor	-0,866451	0,00693	0,0478721
214	CCNA_02449	hypothetical protein	-0,861596	0,0007	0,0067258
215	CCNA_03096	TonB-dependent receptor	-0,859803	7,49E-05	0,0009324
216	CCNA_00947	flagellar hook protein FlgE	-0,852767	0,00641	0,0455141

217	CCNA_01220	serine palmitoyltransferase	-0,850873	0,00047	0,0047632
218	CCNA_00944	flagellar hook length determination protein	-0,849972	0,00017	0,0019573
219	CCNA_03467	hypothetical protein	-0,846304	0,00012	0,0013944
220	CCNA_01181	hypothetical protein	-0,845916	0,00165	0,014189
221	CCNA_00853	3-isopropylmalate dehydrogenase	-0,841324	0,00047	0,0047456
222	CCNA_03286	transporter	-0,840942	0,00054	0,0053743
223	CCNA_01671	diguanylate receptor protein dgrA	-0,839315	0,00062	0,0060466
224	CCNA_03040	outer membrane pilus secretion channel cpaC	-0,838347	0,00016	0,0018647
225	CCNA_03933	hypothetical protein	-0,836738	0,00019	0,0021641
226	CCNA_02645	S-adenosyl-methyltransferase mraW	-0,836346	0,0035	0,0270763
227	CCNA_00028	TonB-dependent receptor	-0,831973	0,00061	0,0060152
228	CCNA_01005	flagellar basal-body rod protein flgC	-0,829987	0,0012	0,0107399
229	CCNA_00952	flagellar assembly protein FlbE/FliH	-0,823626	0,0019	0,0160238
230	CCNA_00425	hypothetical protein	-0,822988	0,00336	0,0261673
231	CCNA_02639	UDP-N-acetylmuramoylalanine--D-glutamate ligase	-0,820277	0,00363	0,0279109
232	CCNA_03531	carboxypeptidase S1	-0,820215	0,0002	0,002232
233	CCNA_03090	acetyl-coenzyme A carboxylase carboxyl transferase subunit alpha	-0,819814	0,00015	0,0016859
234	CCNA_02509	glycosyltransferase hfsG	-0,81892	0,00283	0,0228679
235	CCNA_02961	N-acetylneuraminate synthase	-0,818244	0,00101	0,0093773
236	CCNA_02138	hypothetical protein	-0,814728	0,00131	0,0116178
237	CCNA_02640	phospho-N-acetylmuramoyl-pentapeptide- transferase	-0,814401	0,00037	0,0038455
238	CCNA_02140	flagellar motor switch protein fliM	-0,812975	0,00126	0,0112245
239	CCNA_00080	LexA-related transcriptional repressor	-0,809499	0,00026	0,0028048
240	CCNA_02641	UDP-N-acetylmuramoyl-tripeptide--D-alanyl-D- alanine ligase	-0,808499	0,00026	0,0028554
241	CCNA_02163	hypothetical protein	-0,807182	0,0012	0,0107399
242	CCNA_01117	hypothetical protein	-0,806545	0,00104	0,0095683
243	CCNA_02221	methionine synthase I metH	-0,799147	0,00138	0,0121438
244	CCNA_03121	hypothetical protein	-0,798173	0,00083	0,0078382
245	CCNA_02361	polysaccharide biosynthesis protein celD	-0,79652	0,00108	0,0099077
246	CCNA_02172	transporter	-0,796063	0,00154	0,0133396
247	CCNA_03885	Abi superfamily/CAAX amino terminal protease	-0,794258	0,00296	0,0236377
248	CCNA_02409	hybrid sensor histidine kinase/receiver protein	-0,791648	0,00109	0,0100576

249	CCNA_02144	flagella basal body P ring formation protein flgA	-0,79072	0,00641	0,0455141
250	CCNA_00665	GAF-family sensor histidine kinase	-0,785735	0,00664	0,0466355
251	CCNA_03396	trypsin-like serine protease, typically periplasmic, contains C-terminal PDZ domain	-0,774429	0,00147	0,0128453
252	CCNA_00137	hybrid two-component histidine kinase/receiver protein ShkA	-0,774168	0,0007	0,0067476
253	CCNA_00984	phosphinothricin N-acetyltransferase	-0,774093	0,00083	0,0078318
254	**CCNA_01221**	acyl carrier protein	-0,770191	0,00355	0,0273753
255	CCNA_02222	5-methyltetrahydrofolate--homocysteine methyltransferase homocysteine-binding subunit	-0,76415	0,00078	0,0074088
256	CCNA_R0066	23S RNA	-0,758802	0,00212	0,0176219
257	CCNA_02606	hybrid sensor histidine kinase/receiver domain protein	-0,751589	0,00301	0,0238718
258	**CCNA_00248**	sensor histidine protein kinase	-0,742028	0,00646	0,045581
259	CCNA_02565	hypothetical protein	-0,732791	0,00312	0,024568
260	CCNA_00626	methyl-accepting chemotaxis protein	-0,726941	0,00333	0,0260266
261	CCNA_02061	hypothetical protein	-0,726449	0,0024	0,0197616
262	CCNA_02342	hypothetical protein	-0,712639	0,0014	0,0122451
263	CCNA_03045	TadG-related pilus assembly protein	-0,710191	0,00289	0,0233196
264	CCNA_03034	hypothetical protein	-0,709776	0,00212	0,0176219
265	CCNA_02843	toxin protein parE-3	-0,705253	0,00486	0,0356817
266	CCNA_02507	polyisoprenylphosphate hexose-1-phosphotransferase hfsE	-0,700272	0,00479	0,0353219
267	CCNA_03038	pilus assembly ATPase cpaE	-0,694468	0,00452	0,0336347
268	CCNA_03713	RNA polymerase sigma-54 factor rpoN	-0,693479	0,00163	0,0139929
269	CCNA_01967	hypothetical protein	-0,691658	0,00257	0,021023
270	CCNA_03403	hypothetical protein	-0,689745	0,00434	0,0327285
271	CCNA_02223	beta-lactamase, type II	-0,688964	0,00193	0,0162181
272	CCNA_01193	amylosucrase	-0,683499	0,002	0,0167317
273	CCNA_01165	hypothetical protein	-0,682255	0,00688	0,0476575
274	CCNA_02402	methyl-accepting chemotaxis protein	-0,678785	0,00161	0,0139004
275	CCNA_01530	flagellin	-0,678675	0,00315	0,0247677
276	CCNA_01180	penicillin acylase	-0,67762	0,00273	0,0221159
277	CCNA_00985	protease II	-0,671779	0,00246	0,0202042
278	CCNA_00148	hypothetical protein	-0,670019	0,0038	0,0290334
279	**CCNA_03194**	integral membrane protein	-0,662567	0,00491	0,0359661

280	CCNA_00149	transcriptional regulator, Cro/CI family	-0,660187	0,00461	0,0342187
281	CCNA_01425	H+ translocating pyrophosphatase	-0,645281	0,00329	0,0257277
282	**CCNA_00836**	flagellin	-0,64323	0,00723	0,0492411
283	CCNA_03803	cellulose biosynthesis protein CelD	-0,638073	0,00419	0,031697
284	CCNA_01140	sensory box/GGDEF family protein	-0,637155	0,00578	0,0416967
285	CCNA_00589	hypothetical protein	-0,630634	0,00639	0,0455141
286	CCNA_02343	hypothetical protein	-0,629967	0,0071	0,0485986
287	CCNA_03222	ring hydroxylating dioxygenase, alpha-subunit	-0,628857	0,00547	0,0397004
288	CCNA_03061	3-oxoacyl-(acyl-carrier protein) reductase	-0,62103	0,00472	0,0348693
289	CCNA_01625	aminobenzoyl-glutamate utilization protein B	-0,61917	0,0066	0,0464727
290	CCNA_02944	hypothetical protein	-0,615087	0,00538	0,039262
291	CCNA_00048	S-adenosylmethionine synthetase	-0,607808	0,00541	0,0394684
292	**CCNA_03590**	hypothetical protein	-0,604046	0,00463	0,0343299
293	CCNA_00279	NAD(P)H dehydrogenase (quinone)	-0,600041	0,00549	0,0398095

Genes highlighted in green were identified in the ChIP-seq regulon in this work, and those in yellow the RNA-seq regulon presented by [1].

Supplementary data references.

1. Stein BJ, Fiebig A, Crosson S. The ChvG-ChvI and NtrY-NtrX Two-Component Systems Coordinately Regulate Growth of *Caulobacter crescentus*. J Bacteriol. 2021;203(17):e0019921. Epub 2021/06/15. doi: 10.1128/JB.00199-21. PubMed PMID: 34124942; PubMed Central PMCID: PMCPMC8351639.
