# Supplementary material for: The two-component system ChvGI maintains cell envelope homeostasis in Caulobacter crescentus
Source: PLoS Genet. 2022 Dec 8;18(12):e1010465. doi: 10.1371/journal.pgen.1010465 (PMC9731502; doi:10.1371/journal.pgen.1010465)
Supplement: S3 Table — (PDF) [file pgen.1010465.s010.pdf]

S3 Table. Sequences for ChvI MEME analysis.

	Gene found as positive (+) or negative (-) regulated	Gene identifier and Sequence +/-100bp TSS	TSS obtained from
**1**	+	>CCNA_02075<br>CTCTGCGACGTACGCTTCGTGCCCCTGCTGGCCGGCGTCGCCAAG<br>GATCAGTGAGTTTCT<br>GGGGACGCGTTGATCGCGTCTCAGGATGTTAAAGGTTGGTTTAC<br>CCTGGCGGAGCCCTTT<br>GGAGCTCCGACGGGAAAGGAGACGCTatgAGGCAGTTGTGGACG<br>CAAGCGGCGGTGATCG<br>CTCTGACGGCTGGAACGCTCG	[1]
**2**	+	>CCNA_02817<br>GACAGGAAGCAGGAGCGTGTGGCGTATCGGTGGTCCACGCCGA<br>CGCGGGCGACAGGTCCT<br>GCGTCTCAACAAAGACACAATCTCAAAGTCTACGGCAGGTAGCC<br>ACGCAGCGGCAAAGCG<br>TGCGCTGGCGAAGTGCGCCCCCGAAGCAGCAGGGATCATATCGC<br>GACTTTTTCGGCAACC<br>GTTGCACTGGGTCATCTGCTC	[1]
**3**	+	>CCNA_03091<br>GGGCTGCGAACCTAGAGGCCAAATCCCCGAAGGGAAAGAGGCG<br>AGATTTCGGCCCGGAAC<br>CGCCCTGCTGTCGCCTCGTTTTATCCACAGGATCGATCCCCAGGT<br>GAGTCTCGATTCAAG<br>ACTCTCTTGGTCTGGTGAATCGCGACATAGAGTCCTATGTTGTTTT<br>GTAGCAGGTCGCGG<br>AAGCCGATCTGCATCCCTGCG	[1]
**4**	+	>chvI<br>CGTACGAGACCTTAAGGCGAGGTTGAGGATAGTTTCTGGTGACC<br>CGTGCGCCGCAATCGC<br>GCCTTTCTCGAGCCGCAAAGCGTCGCCAGAAGCAGGGAAAGCTT<br>TACCGCGCCGTGCGTT<br>GAAGCTGTGACGCCCGACCGATAGGATCGGGCGAACACGGAAG<br>GTGAAGGACACGTATCG<br>ACCCGatgGCCGCGATCACGC	[2]
**5**	+	>chvI<br>TGGCGTACGAGACCTTAAGGCGAGGTTGAGGATAGTTTCTGGTG<br>ACCCGTGCGCCGCAAT<br>CGCGCCTTTCTCGAGCCGCAAAGCGTCGCCAGAAGCAGGGAAAG<br>CTTTACCGCGCCGTGC<br>GTTGAAGCTGTGACGCCCGACCGATAGGATCGGGCGAACACGGA<br>AGGTGAAGGACACGTA<br>TCGACCCGatgGCCGCGATCA	[1]
**6**	+	>CCNA_01583<br>CCCCGCCGCCGGAGCCGCGCCCCTGAGGAATGGGTCGAGCTTCG<br>TCGATCGACGGATTTA<br>CTGCCTTAAAACTGTCGTCAAACTGTGACAAAAGCCCGTCATCAG<br>TCTGGCACGCTTCCG<br>TTGCGTCCGGACGAGACAGGATTTGACGACatgAACAAGCTCATC<br>GGCGCGGTCGCCACC<br>GTCGCTCTGCTCGCCGTCGCC	[1]

**7**	+	>CCNA_00735<br>CTTCTGCTCGACAGCTTCCTGTCGGAAGAGAACAAACTGTCGCAT<br>CAGACCGAGTAGTTT<br>TGGCCGATTGCCGCCGCAATCGCGTGGGACATTGGCGCCCGCGC<br>GAGGATGTCGTATCGA<br>GAGCGTCCGAATTTTTGGGCCGGGGGTCGGCCTGCTGGAGCATG<br>GTTTGatgAGTTTCAA<br>CCGGGTCGCCGCGATTAGCGC	[1]
**8**	+	>CCNA_01427<br>AAACGCCTCGATTTCGCCGCGACATCCCGCTATGCGCGGAGTCCG<br>CCTTCACGCGGGCAT<br>TGAACTAAGCCCGCGCGAGCGATAGGTCAAAGTCTGCATTTTTCC<br>GGTTCGGAGCCTTGa<br>tgTCTCGCCCCGCCGTTTTCGCCGCCGCCGTCATGGCCGTGGCGCT<br>CGCTTCTTCGGCGT<br>GCACGCCGCTGACAAGCTATT	[1]
**9**	+	>CCNA_02846<br>CGCCTGAACCGGCGCTGTCGCAGGACGAGGAGGCGCGCGTCCG<br>GCGCCTTCTCGAAGGCG<br>ATTGACACGATTGCGCCGAAATAGCGCCGCAAGAAGCGCCTTCC<br>GGATGATGGAAAGATA<br>ATTCGAGAACCATTGCACCTTTAAATACCGCGACTAGGTTCTCAA<br>AGAGATCGGGGCCGC<br>GCGCCGGGCGCGCGGATGACG	[1]
**10**	+	>CCNA_01759<br>AGACCGCTACGCCTCGACGGCCAGCGGTTTGCGGCTGCAACCTG<br>ATCGTACGATCGTGTT<br>CCGCCTAACGCTCGCCACATTGGGTGATACAGGGTACAGCAGCA<br>ACTAAGTGACCGCGCT<br>TGGGGGCGTGCGGGCGGACCCGCAGGCTTGCCAAGCGTGACTA<br>TCGGGGGCGACGGCCGC<br>AAGGACACGTCGGTCGGACGA	[1]
**11**	+	>CCNA_01090<br>GCTGCGGCCGCGACGCGCGCCTCAAGCAGGTGTGGGGCGGCTA<br>AGTTCGCACTCAACCTA<br>GCCTCGACTCCGCCGCATTCCCGTGTAGCTTACCTCTGTAAATACT<br>CGGGAGGACGTCGa<br>tgAACCGCCGCATGATGCTGGCCTCGGCCGCGCTGCTACTCGCCC<br>CGCTGGCCGCACAGG<br>CGCAGACCCGCGACGTCTCGG	[1]

**12**	+	>CCNA_03357(P1)<br>TCCCTATTTTGCTGCGCGTTTTGCGGGGGAAAGACGCGAAGTCAA<br>GCCCGAGAAACTGAC<br>AACCGTCGTTAGAAAGCTGCTATGGGCTGCGCATCGCCCAATTAT<br>CGCCCCCGCGTCTGG<br>CTTAGACCCGTTCGATCTATATGTTGCGTCGCATGATGGATTTCCG<br>CCGatgATCCCGGC<br>CGACAGTACGGCCCTCGACCT	[1]
**13**	+	>CCNA_03357(P2)<br>ACCGGCGTTCCCTATTTTGCTGCGCGTTTTGCGGGGGAAAGACGC<br>GAAGTCAAGCCCGAG<br>AAACTGACAACCGTCGTTAGAAAGCTGCTATGGGCTGCGCATCG<br>CCCAATTATCGCCCCC<br>GCGTCTGGCTTAGACCCGTTCGATCTATATGTTGCGTCGCATGAT<br>GGATTTCCGCCGatg<br>ATCCCGGCCGACAGTACGGCC	[1]
**14**	+	>CCNA_01505<br>CCAGATGGATTTCGCCCGCGATGTGGCCGAGCGAACGATTTTCAT<br>GGACGGCGGCGTGAT<br>CGTAGAGCAAGGACCTTCGCGTCAGCTCCTCGCCTCGCCGCGCG<br>AAGAGCGGACCCGGCG<br>TTTCTTGGCCAGATCGGGCGTCCTCCAGCCATAATTTGGCCCACG<br>GGACGCTTCACGACC<br>TGAGCGCCGACGCCGGGTGAT	[1]
**15**	+	>CCNA_03341(tolQ)<br>AGCTCTCACCCTGGCTGATCCCGACCGACAGCTGAGCGATGTCGA<br>TTTTCCGGATCGCTC<br>GACGATTGTCATAGTTTCACCGCAGCGCCGCTTCATGCCATACGG<br>CGCTCTCCAGTCCTG<br>ATCAGACGACCCAACGGAGCCTATCCCGAatgGACGCCGCGGCCG<br>CCGCCCCGAATTTCT<br>CGTTCTTCGCCCTGTTCATGC	[1]
**16**	+	>CCNA_03439<br>CTACGCGCTGGGCCAAGCCCAGATTTGAGTGCTGTGCGGAGGTT<br>TTTTCGTTCGGGCGCG<br>GAACAACCGTCAAGGTTCCGTCTTACAACTGCGGCACACTGCCTC<br>CAGACGAACGCCGCg<br>tgGAGACCGACCCGATGCGCATCTTCCAACCGATCATGCTGGTTT<br>GCGCCGTAGCCATGT<br>CGTTCGTCGGCGCCCTGGGCA	[2]

**17**	+	>CCNA_03439<br>CGAGAGGCCTACGCGCTGGGCCAAGCCCAGATTTGAGTGCTGTG<br>CGGAGGTTTTTTCGTT<br>CGGGCGCGGAACAACCGTCAAGGTTCCGTCTTACAACTGCGGCA<br>CACTGCCTCCAGACGA<br>ACGCCGCgtgGAGACCGACCCGATGCGCATCTTCCAACCGATCAT<br>GCTGGTTTGCGCCGT<br>AGCCATGTCGTTCGTCGGCGC	[1]
**18**	+	>CCNA_00733<br>CGAAGGCGGCGTGCTGGATCGCCTGCGGGCCAAGGGCTTCACCG<br>TCACGGCGCCCGAATA<br>AGGTCAAGCTTCCGCCACGCCGCGCGTCCAGGATCGCGCGGCGT<br>TCACAGGGGGATCAAC<br>atgCGCGCGATCTGGGCCGCTCTGGCCATGAGTCTCACCGTCGCC<br>GGCGGGGCCGCCGCC<br>CAGGTGATGGACGATCCGGAG	[1]
**19**	+	>CCNA_00290<br>TCACCAATCTTGGGACTCTGTCGACCACCGGCGTCAATAATGGCG<br>TGGGCGTCCTGATCA<br>TCGGCGGCAGGACCGGCGCCCTGACCAACTCGGCCACGATTTCG<br>CTTCTGGAAGACTACA<br>CCGCCTCTGATAGCGACTCCGACGGCGACCTGGACGGCGTGTTC<br>GCCCAGGGCGCCAACC<br>GCTTTGGCGTTCGCCTGACCA	[1]
**20**	+	>CCNA_00290<br>GCGCCCCGCCAAGCCATGCGATAGACGATTGGCGCCTTAGTGCG<br>TGTCGCGAGCGGGACT<br>CACCGTATTTTCGCCGCATCGTGACCCGAATTAGTGGCCCGGTTT<br>CTATCGAGGTGTCGT<br>TCGTAATGCAGCGCAAGGTCCTGGTCGCGACGGTCGCGGCCGCT<br>CCTCTCCTGGCCATGG<br>GTTTCGCCGCCTCGGCCGAGA	[1]
**21**	+	>CCNA_00290<br>gTTCGCCAGCGGCATCGTGGAGCTTCACCGATTCAAGCGAGAGG<br>CGATTCGACGAAGCAG<br>ATATGGCGCCCCGCCAAGCCATGCGATAGACGATTGGCGCCTTA<br>GTGCGTGTCGCGAGCG<br>GGACTCACCGTATTTTCGCCGCATCGTGACCCGAATTAGTGGCCC<br>GGTTTCTATCGAGGT<br>GTCGTTCGTAATGCAGCGCAA	[1]

**22**	+	>CCNA_00124<br>TGATCACCGCCGCCGGCTTGATCGTCTCGCGCGGCTAAGTTTTCG<br>CCTGTGAGGCAAAGC<br>GGCCttgGCGAAACCACAAAGCCCAGCCAGAGTTAAGCCTGATGT<br>CCAAGGACTCCGCGT<br>GAAGGAGCACGTCATGTCGATCAAGAAGGTCGCCCTCGCCGCCG<br>CCCTGGTGGTGACCGC<br>CGGTCTCGCCGCCTGTGCGAC	[1]
**23**	+	>CCNA_01660<br>CACGATCCGGCGGGGACCGGTGGAAGCGGCGCGGAACATAGTC<br>GGGGGTCGCCTGCGTCG<br>CAAGCACGGTTTCGCCGTTTTTGCGTGGGATGACGGTAAAGCTGT<br>GTACGCGTTAGGGTG<br>ACTCatgCGTCGAACCTTGCTTTTGGCGGCCTTTTGCGGCGCGACG<br>CTCGCCGCGAGCGC<br>TTCCGCGCGCGCTCAACAAGC	[1]
**24**	+	>CCNA_01067<br>GCGACGCGGCGCGCAGGCTATGGCGCGACAAGAATGTCACATG<br>GGAAGAGACGGTCCGCC<br>GTCTTCTTGATTAAGCCACAAACATTGGGTTGAAGACCACGTTAA<br>GACGGGGTCGGCTAC<br>AGTCTAGGAAatgCGAGTGCTGTCGAAAGTTCTGTCCGTGCGAAC<br>GTCTCTGATCGCCTT<br>GGCCATGGCCATGGCGGTCGT	[1]
**25**	+	>CCNA_01087<br>ACATTTGCGCCGCTTCGCGAGGGGCGTTGCGGTCGCGGTGGGGG<br>CGTGGCGCGCGGTTAT<br>GCTTAACCACGCGTTAAGTTTGGAGCCCCAAACCGGACGCAATG<br>GCTTGCAACTGTGTTG<br>CAAACAATTCCATCCGGAGGCCCTGACATGGACCGTCGCAAGCT<br>GATCCTCTCGAGCGCC<br>GCCCTGGGCGTGTCCGCTGTG	[1]
**26**	+	>algH<br>GCGACTCGAATAGACCCCCGCGCGGCGGCCGCCAAGCACGCTTT<br>GATGAAGCATCCGACT<br>TGAAACCGTCACAACGAACGCCAATGCTTGAGGTatgGCCAGCTT<br>AATCGACGATGACGA<br>CGGCGAGTTCCTGATCGGCAGGCTGCTCGTCGCCATGCCCGGCA<br>TCGAGGATCCGCGCTT<br>CGAGCGGACGGTGCTGTATCT	[2]

**27**	+	>algH<br>ATTGAGGCGACTCGAATAGACCCCCGCGCGGCGGCCGCCAAGCA<br>CGCTTTGATGAAGCAT<br>CCGACTTGAAACCGTCACAACGAACGCCAATGCTTGAGGTatgGC<br>CAGCTTAATCGACGA<br>TGACGACGGCGAGTTCCTGATCGGCAGGCTGCTCGTCGCCATGC<br>CCGGCATCGAGGATCC<br>GCGCTTCGAGCGGACGGTGCT	[1]
**28**	+	>CCNA_03601<br>GTTCGACGGCGCCCGCGGCCTATTGACCTGATCGCGTTTTTCCAC<br>GCGTTCGTGTCGCCT<br>CGGCCCTTTCGCGGCCATGATGATCGACGCTTATATGTCGCGGAA<br>CGAACCTATGCTTCG<br>CGCATACTGCAAGCCACCAGAGCCGGGCCatgACCGAACTCACCA<br>TCACCAGCCAAGCGA<br>CCGACTTGGCCATCGCCAAGC	[1]
**29**	+	>CCNA_03601<br>TGTTCGACGGCGCCCGCGGCCTATTGACCTGATCGCGTTTTTCCA<br>CGCGTTCGTGTCGCC<br>TCGGCCCTTTCGCGGCCATGATGATCGACGCTTATATGTCGCGGA<br>ACGAACCTATGCTTC<br>GCGCATACTGCAAGCCACCAGAGCCGGGCCatgACCGAACTCACC<br>ATCACCAGCCAAGCG<br>ACCGACTTGGCCATCGCCAAG	[1]
**30**	+	>CCNA_01653<br>GGCTAGAGCCCCTTCCTATCCGATAACTGTTTCACACTTATCGGAA<br>AATGCTCCAGGCTT<br>CGCCGTTATCCGGCCGAATTCCTGCGCTTCAACGACCGTCGCACT<br>TGTCGGGGAGGCCGT<br>GGCGTTCTACGCCCGGCCGCGACTGTTCTGTTCGGGGTACTTCatg<br>AAGCTCGCCACGAC<br>CGCCGCCGCGCTGGCGCTGGC	[1]
**31**	+	>CCNA_03838<br>CGTGACCCTGGGCCCCTGGGCGGCCACCGGCCCGGCCAGCAAAG<br>CCGCTCCCGCCGCAAT<br>TCCGACCATGACACGCGTCAAACCCATTCGAGACGAACCTTGTTC<br>TGTTTTTGACCGCAT<br>GTTGCTTGGCCCCCACCGGCCCCTttgCTACAGCTTTCAAACGCCT<br>GTCAGAGAGAGCTT<br>TTCAAACCGCCCATGGCCGCC	[1]

**32**	+	>CCNA_03726<br>ATGGATGTCTCGATAGGCGCCCCCGTGGGCCGAGGTCAACGACA<br>CGACGCGATCAGGGTT<br>CCGCTTGCCTGGGCCGGGAAACAGTCGGTTACATCAATTGGCTC<br>GAAATCGTCACGAAGC<br>CTCACTAAGAGCGCCGCCCatgAGCATGCCCTTCATCGACCTCGGC<br>GCGCAGCAGCGCCG<br>GATCCGCGACAAGATCGACAC	[2]
**33**	+	>CCNA_03726<br>ACACGACGCGATCAGGGTTCCGCTTGCCTGGGCCGGGAAACAGT<br>CGGTTACATCAATTGG<br>CTCGAAATCGTCACGAAGCCTCACTAAGAGCGCCGCCCatgAGCA<br>TGCCCTTCATCGACC<br>TCGGCGCGCAGCAGCGCCGGATCCGCGACAAGATCGACACGGCC<br>ATCGCCAAGGTGCTGG<br>ACAGCGGCGCCTATGTGATGG	[1]
**34**	-	>phyR<br>ACAACGTGGTTTTCCGTACCCGGTTCCATAAGCTTGGGCATAAGA<br>TGGCAAAATTCTGCT<br>TAGGGCGGAACCGTCGAGTTGAGCGGGCGTTATCGACCTTCGAC<br>GGAACGGCGTAGCTTC<br>GCCGTTAGTTAGTAGATCTTCGAAGACACAGAATCGGGAGGGGT<br>CatgAGTCTTCTTGCT<br>CGCTTGGCGCCGCATTTGCCT	[2]
**35**	-	>phyR<br>AGTTCATCGGCGCCCGCCCCCTTTGGCCGAATGTCTTTCCGCCAG<br>ACGACAACGTGGTTT<br>TCCGTACCCGGTTCCATAAGCTTGGGCATAAGATGGCAAAATTCT<br>GCTTAGGGCGGAACC<br>GTCGAGTTGAGCGGGCGTTATCGACCTTCGACGGAACGGCGTAG<br>CTTCGCCGTTAGTTAG<br>TAGATCTTCGAAGACACAGAA	[1]
**36**	-	>phyR<br>CAACGTGGTTTTCCGTACCCGGTTCCATAAGCTTGGGCATAAGAT<br>GGCAAAATTCTGCTT<br>AGGGCGGAACCGTCGAGTTGAGCGGGCGTTATCGACCTTCGACG<br>GAACGGCGTAGCTTCG<br>CCGTTAGTTAGTAGATCTTCGAAGACACAGAATCGGGAGGGGTC<br>atgAGTCTTCTTGCTC<br>GCTTGGCGCCGCATTTGCCTT	[1]

**37**	-	>phyR<br>GGGCATAAGATGGCAAAATTCTGCTTAGGGCGGAACCGTCGAGT<br>TGAGCGGGCGTTATCG<br>ACCTTCGACGGAACGGCGTAGCTTCGCCGTTAGTTAGTAGATCTT<br>CGAAGACACAGAATC<br>GGGAGGGGTCatgAGTCTTCTTGCTCGCTTGGCGCCGCATTTGCCT<br>TACATCCGCCGCTA<br>CGCCCGGGCCTTGACCGGCGA	[1]
**38**	-	>CCNA_00438<br>AAAGTAAACCCGCAATCTTACCGTGCGACTCAGAATATCTTAACT<br>TGCACCTGCGAATtt<br>gCTCCTGCGTCCAGTTGGCGCGTCGTCCACAGGACGTGAATTCAT<br>TACTAGGAGCAGCGG<br>GATGAAATATCCGATCGCTATTGCCGCGGGAATCGCTGGCAGCTT<br>TTTTGTCGCTCAGGC<br>CGCTTCGGCCGGCGGCGGTTT	[1]
**39**	-	>CCNA_00438<br>GAGAGTCACCAAAGCGGCAGATCGTGGTTACCTGAAAGTAAACC<br>CGCAATCTTACCGTGC<br>GACTCAGAATATCTTAACTTGCACCTGCGAATttgCTCCTGCGTCCA<br>GTTGGCGCGTCGT<br>CCACAGGACGTGAATTCATTACTAGGAGCAGCGGGATGAAATAT<br>CCGATCGCTATTGCCG<br>CGGGAATCGCTGGCAGCTTTT	[1]
**40**	-	>CCNA_01303<br>CGTACGGACGTGAAGGTCGAGTTCCTGACCACCAACGCCGCTTTG<br>CCGCTCACGGTCCGC<br>CAGGACGGCGACCAGGTGATCGTTGACGGCGACCTTAAGATGAA<br>CCGGATCAAAGGCTGC<br>AATAGCCGCAATGGCAAGATCTGGGTCAAGGTGCGTGGCGTTGG<br>GGATGTCTCCTACGAC<br>AACATTCCTGAGATCGCGGTC	[1]
**41**	-	>CCNA_00974<br>GTTCCTCCTGTGGCGATTTTGCGACAGGGGGGTCGGGATAATGA<br>TTACTTTTTTGCAATC<br>AGAATTGACCCTCGCCCGATCCACCCCCTAACGTCGCTGCAACCG<br>GAAGAGTTTGTTCCG<br>GGACACATGTGATCGCGTGGTGGATTTACAGCGGATTTCGGCTC<br>GAAAACGGACAGGTCG<br>CTGAGGGGCTTCTTGTTCGGC	[1]

**42**	-	>CCNA_00974<br>TCGAGACCTCCACGGCGACCACCGGCGGCACCTATGTTCAGGAC<br>CGCACCGCCGATCCCT<br>TCTACGCCTTCAAGCTTGACGGCTACATCACCGACAAGCACCACC<br>TCGAACTGACCTATT<br>TCGACACCAGCCGTAAGCGCAAGCGCGACCAGTTTACGTTCAACC<br>CGGCCACTGACGTCA<br>TCAGCCCGACGCGCGCCAGCG	[1]
**43**	-	>CCNA_00974<br>ACGGCGACCACCGGCGGCACCTATGTTCAGGACCGCACCGCCGA<br>TCCCTTCTACGCCTTC<br>AAGCTTGACGGCTACATCACCGACAAGCACCACCTCGAACTGACC<br>TATTTCGACACCAGC<br>CGTAAGCGCAAGCGCGACCAGTTTACGTTCAACCCGGCCACTGAC<br>GTCATCAGCCCGACG<br>CGCGCCAGCGCCGAAAGCCTG	[1]
**44**	-	>CCNA_00974<br>CGCACCACATCCGCTTCGGCTACGATCAGGAGAACACCCTCCTGA<br>CGCAGTTCACGCGCC<br>GTAACGGTGGCCGTAACTACACCTACCGTCGTGCAGGCGCTGCG<br>GGTGCTCTGGGCGGGG<br>CCGTGGCCGCGGGTCAAGAGTACATCGAAGGCCGCAAGTTCCAG<br>ACCGGCGGTGGTTTCG<br>CCGGCAAGAACAAGGCCTATT	[1]
**45**	-	>CCNA_03108<br>CCGGGCCACCTCGGGTAAGTCGGTGCGTGCGCCGACCCTGGGTG<br>AGACCTTCCGTCCGCC<br>GACCCAGACCTTCGCCACGATCACCGATCCGTGCGACGCTCGCGT<br>CCTGATCAACCTGAC<br>CGACGCCAAGATCAAGGCGAACCGTGAGAAGAACTGCGCCGCCC<br>TGGGCATCCCGGCCGG<br>CACGAACATCATCTACACGAG	[1]
**46**	-	>CCNA_03108<br>CGGCCCGGCGCGCAGCCAGCTGAACGAAAAGGAACTGAAGCGC<br>TTCGTCGCCGGCTTCCG<br>CGGCAGCAAGGACGACTTCGGCTTCATCCACAACCTGTCGTACGA<br>GCTGGGCTACACCTA<br>CGGCGAGCTGAAGAACAAGAACTTCGAGCGGGGCGTGGATGCG<br>CCGCGCTACAAGTTCGC<br>CGCCGACGCCGTCGTGGACAC	[1]

**47**	-	>CCNA_03108<br>GCGCCAGATACGTGACTTTTTGGTAACGCAACGCCTTCAACCCGT<br>CCGAATTTGGCCATG<br>ATCGCTTCTAGCGCGGCCGAAAGGCTTTAACGTCAGTTTTGAGGA<br>ATGGCCCTCTttgCT<br>CCCGCGCGCGGCTGGCGTGACGGGCGCGCGTACTTTTGAGGACA<br>ACAAAAACATGGCGTT<br>GAAAACCAAGGTTCTCCTGGC	[1]
**48**	-	>CCNA_02200<br>TCTCGAGCTATGCCTGGAGGCGCTGGCCCTCTACGCGGAATAGC<br>GCGCCCGACTTGATCT<br>GCGTCAAGGTGGACGGGCTGGCCGGCCGCATGTTGTCGATCCAG<br>CttgGAGGGGTGGCCG<br>ATGCGTACTGAGATTCGCTGGAGCGTCGTGGTGCTCGGCGCCGT<br>CATGGCTTTCGCGACG<br>ACGAGCCAGGCGGCGCAGGAT	[1]
**49**	-	>CCNA_03325<br>ACAAAACAACGCTAACGCACATTTTTGCGTTGCCTTCGCTTTAACA<br>TTTCGTTTACCTTT<br>TTTGTTAGTATTTCATTAACTGGAGGCGGGCgtgGAAAAGGTCTTT<br>GTTGCGAAGCGCGT<br>GGCGAACAAGCTGTTTGCGACGGAAGCGGCTGTTGACGCGGCG<br>GTGGCCGAAGTCAGCGA<br>GATGATGGCTGAGCTGATGCA	[1]
**50**	-	>CCNA_03325<br>ACGCTTCGCTTCCATCCGAAAGGAGAGTTGCGCGACAAAACAAC<br>GCTAACGCACATTTTT<br>GCGTTGCCTTCGCTTTAACATTTCGTTTACCTTTTTTGTTAGTATTT<br>CATTAACTGGAGG<br>CGGGCgtgGAAAAGGTCTTTGTTGCGAAGCGCGTGGCGAACAAG<br>CTGTTTGCGACGGAAG<br>CGGCTGTTGACGCGGCGGTGG	[1]
**51**	-	>CCNA_01269<br>AACCCTTATAAAATCTGGGCCTGAGGCTTGTCACGTAACAATGGT<br>CCTTAAGCGTGGGGG<br>TTACGCCAAGGCTGCGACAATTGCTGGTATCTACGTGATTCTCAT<br>GGCGAAAAGTGAACT<br>TGCAAAACGTGGGTCTGCACATAACTGTTTGACGGTCGTTAACCA<br>TAAATCTTACCCCTT<br>TTCTCACGGAGCGGGCTGGCT	[1]

**52**	-	>CCNA_01269<br>GGTATCTACGTGATTCTCATGGCGAAAAGTGAACTTGCAAAACGT<br>GGGTCTGCACATAAC<br>TGTTTGACGGTCGTTAACCATAAATCTTACCCCTTTTCTCACGGAG<br>CGGGCTGGCTCCGG<br>GCAGGGCTGGATGCAAGATCatgGACTGGAGCGAAGAACGGACC<br>GCGACGCTGAAGAAGC<br>TGTGGCTGGAGGGCCTGAGCG	[1]
**53**	-	>CCNA_00417<br>ATCGATCCGAGGAGATGACTTTTTTGTAAGGTCCGGCGGACGCG<br>CCCAACGTGACATCGC<br>CTTCatgACGCCACCTTCATTTGCATCACGGCCTGATCAGCGCGAC<br>GATCTGGATAACAA<br>GACTATTCCAGGCTCAACCGTGACCGACCCCACCCCAGACACCGC<br>CGTCCCGCCGACGCT<br>GCAGGACGACAAGGCCCTGCC	[1]
**54**		>CCNA_00166<br>GAGTTCTGTTTTGGTAAATATCGAATTTAGGACAGTTAAACACAT<br>GGATTATACCATCTA<br>AATTCAACTCGTTTTCGGACCTCACATGAATACTGACCTCACGAAC<br>ACAGCGAGGTCAGC<br>atgTTCAAGGCGCGAAACATTGTTATGGGACTGCTCAGCGCCGCT<br>GCGCTGATGACGAGC<br>GCCCAGGCACAGGCCGGCGGG	[1]
**55**		>CCNA_00982<br>GCGATCAACACGCCGCAATTGTTACAACAATTCACGAAGCAAGTT<br>TGATTTCAGTTTCAA<br>ACTACTTGTTTTTCTGAGGCGACCGTGTAATCCCGGATACAAGGG<br>GAACGGAGTTTTGAA<br>CgtgGAAGATAAAGCTACCCTAATCGAGCTTACCGCTGAAATCGTC<br>GCTAACTATGTGGC<br>CAATAATTCGACGCCAGTGTC	[1]

Supplementary data references.

1. Zhou B, Schrader JM, Kalogeraki VS, Abeliuk E, Dinh CB, Pham JQ, et al. The global regulatory architecture of transcription during the *Caulobacter* cell cycle. PLoS Genet. 2015;11(1):e1004831. Epub 2015/01/09. doi: 10.1371/journal.pgen.1004831. PubMed PMID: 25569173; PubMed Central PMCID: PMCPMC4287350.

2. McGrath PT, Lee H, Zhang L, Iniesta AA, Hottes AK, Tan MH, et al. High-throughput identification of transcription start sites, conserved promoter motifs and predicted regulons. Nat Biotechnol. 2007;25(5):584-92. Epub 2007/04/03. doi: 10.1038/nbt1294. PubMed PMID: 17401361.
