# Supplementary material for: The two-component system ChvGI maintains cell envelope homeostasis in Caulobacter crescentus
Source: PLoS Genet. 2022 Dec 8;18(12):e1010465. doi: 10.1371/journal.pgen.1010465 (PMC9731502; doi:10.1371/journal.pgen.1010465)
Supplement: S4 Table — (PDF) [file pgen.1010465.s011.pdf]

S4 Table. Bacterial strains.

Name	Genetic Information	Reference
	**Bacteria**	
Caulobacter crescentus		
WT	NA1000, laboratory strain	
Δ*chvI*	NA1000 Δ*chvI*	This study
Δ*chvG*	NA1000 Δ*chvG*	This study
Δ*chvIG*	NA1000 Δ*chvIG*	This study
Δ*chvT*	NA1000 Δ*chvT*	This study
Δ*chvI* Δ*chvT*	NA1000 Δ*chvI*, Δ*chvT*	This study
Δ*ntrX*	NA1000 Δ*ntrX*	This study
Δ*chvI* Δ*ntrX*	NA1000 Δ*chvI*, Δ*ntrX*	This study
Δ*sigT*	NA1000 Δ*sigT*	This study
Δ*xylX*	NA1000 Δ*xylX*	[1]
$chvI_{D52E}$	NA1000 $chvI_{D52E}$	This study
$chvI_{D52A}$	NA1000 $chvI_{D52A}$	This study
$chvG_{H309N}$	NA1000 $chvG_{H309N}$	This study
Δ*chvI chvI*	NA1000 Δ*chvI* P_{xylX}::*chvI*, Tet^R	This study
Δ*chvI* $chvI_{D52E}$	NA1000 Δ*chvI* P_{xylX}::$chvI_{D52E}$, Tet^R	This study
Δ*chvI* $chvI_{D52A}$	NA1000 Δ*chvI* P_{xylX}::$chvI_{D52A}$, Tet^R	This study
Δ*chvI chvI* Δ*xylX*	NA1000 Δ*chvI* P_{xylX}::*chvI* Δ*xylX*, Tet^R	This study
Δ*chvIG chvI* Δ*xylX*	NA1000 Δ*chvIG* P_{xylX}::*chvI* Δ*xylX*, Tet^R	This study
WT P_{dipM}	NA1000 pMR15-P_{dipM}	This study
Δ*chvI* P_{dipM}	NA1000 Δ*chvI* pMR15-P_{dipM}	This study
WT P_{ftsN}	NA1000 pMR15-P_{ftsN}	This study
Δ*chvI* P_{ftsN}	NA1000 Δ*chvI* pMR15-P_{ftsN}	This study
WT P_{nepR}	NA1000 pMR15-P_{nepR}	This study
Δ*chvI* P_{nepR}	NA1000 Δ*chvI* pMR15-P_{nepR}	This study
WT P_{phyR}	NA1000 pMR15-P_{phyR}	This study
Δ*chvI* P_{phyR}	NA1000 Δ*chvI* pMR15-P_{phyR}	This study
Δ*chvI chvI-egfp*	NA1000 Δ*chvI* P_{xylX}::*chvI-egfp*, Kan^R	This study

Δ*chvI egfp-chvI*	NA1000 Δ*chvI* P_{xylX}::*egfp-chvI*, Kan^R	This study
Δ*chvG chvG-egfp*	NA1000 Δ*chvG* P_{xylX}::*chvG-egfp*, Kan^R	This study
Δ*chvG egfp-chvG*	NA1000 Δ*chvG* P_{xylX}::*egfp-chvG*, Kan^R	This study
Δ*chvG chvG-mcherry*	NA1000 Δ*chvG* P_{xylX}::*chvG-chy*, Tet^R	This study
Δ*chvG* $chvG_{1\text{-}114}$*-mcherry*	NA1000 Δ*chvG* P_{xylX}:: $chvG_{1\text{-}114}$*-chy*, Tet^R	This study
Δ*chvG* $chvG_{1\text{-}274}$*-mcherry*	NA1000 Δ*chvG* P_{xylX}:: $chvG_{1\text{-}274}$*-chy*, Tet^R	This study
Δ*chvG* $chvG_{273\text{-}534}$*-mcherry*	NA1000 Δ*chvG* P_{xylX}:: $chvG_{273\text{-}534}$*-chy*, Tet^R	This study
Escherichia coli		
MT607	*pro*-82 *thi*-I *hsdR17* (r-m+) *supE44 recA56*	[2]
Top10	*F- mcrA Δ(mrr-hsdRMS-mcrBC) φ80lacZΔM15 ΔlacX74 nupG recA1 araD139 Δ(ara-leu)7697 galE15 galK16 rpsL*(Str^R) *endA1* λ^-	Life technology

Supplementary data references.

1. Stephens C, Christen B, Fuchs T, Sundaram V, Watanabe K, Jenal U. Genetic analysis of a novel pathway for D-xylose metabolism in Caulobacter crescentus. J Bacteriol. 2007;189(5):2181-5. Epub 2006/12/19. doi: 10.1128/JB.01438-06. PubMed PMID: 17172333; PubMed Central PMCID: PMCPMC1855722.
2. Casadaban MJ, Cohen SN. Analysis of gene control signals by DNA fusion and cloning in Escherichia coli. J Mol Biol. 1980;138(2):179-207. Epub 1980/04/01. doi: 10.1016/0022-2836(80)90283-1. PubMed PMID: 6997493.
