# Supplementary material for: The two-component system ChvGI maintains cell envelope homeostasis in Caulobacter crescentus
Source: PLoS Genet. 2022 Dec 8;18(12):e1010465. doi: 10.1371/journal.pgen.1010465 (PMC9731502; doi:10.1371/journal.pgen.1010465)
Supplement: S5 Table — (PDF) [file pgen.1010465.s012.pdf]

S5 Table. Plasmids

Plasmids	
**Name**	**Reference**
pNPTS138	M. R. Alley, Imperial College London (UK), unpublished
pNPTS138-Δ*chvI*	This study
pNPTS138-Δ*chvG*	This study
pNPTS138-Δ*chvIG*	This study
pNPTS138-$chvI_{D52E}$	This study
pNPTS138-$chvI_{D52A}$	This study
pNPTS138-$chvG_{H309N}$	This study
pNPTS138-Δ$chvG_{1-274}$	This study
pNPTS138-Δ$chvG_{274-534}$	This study
pNPTS138-Δ*chvT*	This study
pNPTS138-Δ*ntrX*	This study
pNPTS138-Δ*sigT*	This study
pMR15	[1]
pMR15-P_{dipM}	This study
pMR15-P_{ftsN}	This study
pMR15-P_{nepR}	This study
pMR15-P_{phyR}	This study
pET-28a	Novagen
pET-28a-*chvI*	This study
pXC5	[2]
pXC5-*chvI*	This study
pXC5-$chvI_{D52A}$	This study
pXC5-$chvI_{D52E}$	This study
pXGFPC-2	[2]
pXGFPC-2 *chvI*	This study
pXGFPC-2 *chvG*	This study
pXGFPC-2 $chvG_{H309N}$	This study

pXGFPN-2	[2]
pXGFPN-2 *chvI*	This study
pXGFPN-2 *chvG*	This study
pXCHYC-5	[2]
pXCHYC-5 *chvG*	This study
pXCHYC-5 $chvG_{1\text{-}114}$	This study
pXCHYC-5 $chvG_{1\text{-}274}$	This study
pXCHYC-5 $chvG_{273\text{-}534}$	This study

Supplementary data references.

1. Gober JW, Shapiro L. A developmentally regulated Caulobacter flagellar promoter is activated by 3' enhancer and IHF binding elements. Mol Biol Cell. 1992;3(8):913-26. Epub 1992/08/01. doi: 10.1091/mbc.3.8.913. PubMed PMID: 1392079; PubMed Central PMCID: PMCPMC275648.

2. Thanbichler M, Iniesta AA, Shapiro L. A comprehensive set of plasmids for vanillate- and xylose-inducible gene expression in Caulobacter crescentus. Nucleic Acids Res. 2007;35(20):e137. Epub 2007/10/26. doi: 10.1093/nar/gkm818. PubMed PMID: 17959646; PubMed Central PMCID: PMCPMC2175322.
