# Supplementary material for: The two-component system ChvGI maintains cell envelope homeostasis in Caulobacter crescentus
Source: PLoS Genet. 2022 Dec 8;18(12):e1010465. doi: 10.1371/journal.pgen.1010465 (PMC9731502; doi:10.1371/journal.pgen.1010465)
Supplement: S6 Table — (PDF) [file pgen.1010465.s013.pdf]

S6 Table. Oligos

ID	Sequence
592	cttagtcaagcttcttgaagccatgcccgtcac
648	cgacgaaaccgatcggatcc
649	cttagtcgaattcgaggacgagacggatagagc
650	cttagtcgaattcttgggcctcaaccgcaacac
651	cttagtcaagcttcgacgtaggaattggcgacc
1013	cgggaatggcgatcttctgc
1014	tcgaattcgtcaatgagcgtgatcgcg
1015	tcaggtcgacccggaattcg
1016	tcaagcttgctgggcggcgataatctcg
2055	cctaagtaactaaggatcctagtggtcgccggtcgcttg
2056	cctaagtaactaagaattcgtccgacctgttccgcgac
2057	cctaagtaactaagaattcagcgattgagcgccttgcgg
2058	cctaagtaactaaaagcttcgtcggcatagaccacgc
2155	ttagttacttaggcatatggctaccgttatcgcgaagcc
2156	cctaagtaactaagagctcgcgaaagtcacgacgcgatc
2157	ttagttacttaggcatatggccgcgatcacgctcattga
2158	cctaagtaactaagagctccgcagctcgttgaggatcaa
2173	ttagttacttaggggatcccgacctccttcttgggcttgc
2174	cctaagtaactaagaattcgcgagaccgaagcgacgatg
2177	aactaaggatcctcagacgtccgagatccctg
2179	aactaaggtaccagaaccgcaaggacgcgaac
2180	tcctaagtaactaaaagcttacgagcccgatcatgccttg
2405	ggatccccgggtacatatgatggccgcgatcacgctcatt
2406	cctaagtaactaaggtacctcaggcttcgcgataacggtagc
2411	cctaagtaactaaggtacccaggcttcgcgataacggta
2563	ttagttacttagggaattcagaaccgcaaggacgcgaac
2568	ggtagaattctcgcgcgcgtccggcaattcga
2569	aactaaggtaccttggctaccgttatcgcgaagcc
2570	aactaaggtaccgccgcgatcacgctcattgacg
2576	cttcgccgccgacgttgcgaacgagatcaagaatccgct
2577	agcggattcttgatctcgttcgcaacgtcggcggcgaag
2578	gtcacggccgaagctagctctgttgttcattccgcgctcg

2579	tgcaggatatctggatccactgggtctcgacgatctgacgggcgatcg
2580	ggacctagtgatcctggccgtgaagatgccgcgcatgga
2581	tccatgcgcggcatcttcacggccaggatcactaggtcc
2582	ggacctagtgatcctggaagtgaagatgccgcgcatgga
2583	tccatgcgcggcatcttcacttccaggatcactaggtcc
2584	gtcacggccgaagctagctagtttctggtgacccgtgc
2585	tgcaggatatctggatccacaacggtagccaacgccgtacag
2773	gtaactaaggtaccgttccgagaggtcgggaagc
2870	tcacggccgaagctagcgaattcgtggatcaccgaggatcacagagggtc
2871	gaggaagaaacgacggttgtcctcaaaagtacgcgc
2872	acttttgaggacaaccgtcgtttcttcctcggct
2873	ggcccgtgcaattgaagccggctggcgccaaagctgttgaggacgagatcgc
3131	cttagggaattctccgagaggtcgggaagcgag
3132	gtaactaacatatgtcggaacggaaggacgaactg
3133	cttagggaattctgggcgtcgaacagccgcg
3135	gtaactaaggtaccaggcttcgcgataacggtagc
3136	gtaactaaggtacctcggaacggaaggacgaactgg
3170	ttagttacttaggtctagagtgtagagatcggcgggctg
3171	cctaagtaactaagaattccgggtcgatacgtgtccttcac
3174	ttagttacttaggtctagacgggtgctggagatcggcac
3175	cctaagtaactaactcgagagcgtctcctttcccgtcggag
3176	cctaagtaactaactcgagagcgtctcctttcccgtcggag
3177	cctaagtaactaactcgagctcagctaggctccaaaactcgg
3182	ttagttacttaggtctagaggcgtagcggcggatgtaagg
3183	cctaagtaactaactcgaggtcctcgacgccgaagttcatcgg
3184	ttagttacttaggtctagattcatccagcgccgcagcgc
3185	cctaagtaactaactcgaggacccctcccgattctgtgtc
