# Supplementary material for: The two-component system ChvGI maintains cell envelope homeostasis in Caulobacter crescentus
Source: PLoS Genet. 2022 Dec 8;18(12):e1010465. doi: 10.1371/journal.pgen.1010465 (PMC9731502; doi:10.1371/journal.pgen.1010465)
Supplement: S1 Text — Construction of plasmids. (PDF) [file pgen.1010465.s014.pdf]

S1 Text. Construction of plasmids

pNPTS138-Δ*chvI*
Upstream and downstream regions of *C. crescentus chvI (CCNA_00237)* were amplified from WT gDNA by PCR respectively with primers 1013/1014 (690 bp) and 1015/1016 (710 bp). The PCR were then respectively digested with *Bam* HI/*Eco* RI and *Eco* RI/*Hind* III; and ligated into the pNPTS138 vector cut with *Hind* III and *Bam* HI.

pNPTS138-Δ*chvG*
Upstream and downstream regions of *C. crescentus chvIG (CCNA_00237-CCNA_00238* were amplified from WT gDNA by PCR respectively with primers 2173/2174 and 2563/2180. The PCR were then respectively digested with *Bam* HI/*Eco* RI and *Eco* RI/*Hind* III; and ligated into the pNPTS138 vector cut with *Hind* III and *Bam* HI

pNPTS138-Δ*chvIG*
Upstream and downstream regions of *C. crescentus chvIG (CCNA_00237-CCNA_00238* were amplified from WT gDNA by PCR respectively with primers 2173/2174 and 2179/2180. The PCR were then respectively digested with *Bam* HI/*Eco* RI and *Eco* RI/*Hind* III; and ligated into the pNPTS138 vector cut with *Hind* III and *Bam* HI.

pNPTS138-Δ$chvG_{1\text{-}274}$
Upstream and downstream regions of *C. crescentus* $chvG_{1\text{-}274}$ *(CCNA_00237)* were amplified from WT gDNA by PCR respectively with primers 2173/3135 and 3136/2180. The PCR were then respectively digested with *Bam* HI/*Kpn* I and *Kpn* I/*Hind* III; and ligated into the pNPTS138 vector cut with *Hind* III and *Bam* HI.

pNPTS138-Δ$chvG_{274\text{-}534}$
Upstream and downstream regions of *C. crescentus* $chvG_{274\text{-}534}$ *(CCNA_00237)* were amplified from WT gDNA by PCR respectively with primers 2177/2773 and 2179/592. The PCR were then respectively digested with *Bam* HI/*Eco* RI and *Eco* RI/*Hind* III; and ligated into the pNPTS138 vector cut with *Hind* III and *Bam* HI.

pNPTS138-Δ*chvT*
Upstream and downstream regions of *C. crescentus chvT (CC3013*) were amplified from WT gDNA by PCR respectively with primers 2870/2871 and 2872/2873. The PCR products were then assembled with the pHR253 (pNPTS138) vector cut with *Bam* HI and *Hind* III via Gibson assembly.

pNPTS138-Δ*ntrX*
Upstream and downstream regions of *C. crescentus ntrX (CC1743*) were amplified from WT gDNA by PCR respectively with primers 648/649 (620 bp) and 650/651 (580 bp) and cloned into pSK. The pSK-648/649 and pSK-650/651 recombinant plasmids were then digested respectively with *Hind* III/*Eco* RI and *Eco* RI/*Bam* HI; and ligated into the pNPTS138 vector cut with *Hind* III and *Bam* HI.

pNPTS138-Δ*sigT*
Upstream and downstream regions of *C. crescentus CC3475* were amplified from WT gDNA by PCR respectively with primers 2055/2056 and 2057/2058. The PCR were then respectively digested with *Bam* HI/*Eco* RI and *Eco* RI/*Hind* III; and ligated into the pNPTS138 vector cut with *Hind* III and *Bam* HI.

pNPTS138-$chvI_{D53A}$
Upstream and downstream regions of *C. crescentus chvI (CC0237* or *CCNA_00237)* were amplified from WT gDNA by PCR respectively with primers 2584/2581 and 2580/2585. A single PCR fragment was generated by overlap extension PCR. Thereafter, the PCR fragment was cloned using blunt ligation with pNPTS138 vector cut previously with EcoRV.

pNPTS138-$chvI_{D53E}$
Upstream and downstream regions of *C. crescentus chvI (CC0237* or *CCNA_00237)* were amplified from WT gDNA by PCR respectively with primers 2584/2583 and 2582/2585. A single PCR fragment was generated by overlap extension PCR. Thereafter, the PCR fragment was cloned using blunt ligation with pNPTS138 vector cut previously with EcoRV.

pNPTS138-$chvG_{H309N}$
Upstream and downstream regions of *C. crescentus chvI (CC0237* or *CCNA_00237)* were amplified from WT gDNA by PCR respectively with primers 2577/2578 and 2576/2579. A single PCR fragment was generated by overlap extension PCR. Thereafter, the PCR fragment was cloned using blunt ligation with pNPTS138 vector cut previously with EcoRV.

pXC5-*chvI*
chvI was amplified from WT gDNA by PCR primers 2405/2406. The PCR was then digested with *Nde* I and *Kpn* I, and ligated into the pXC-5 vector cut with the same restriction enzymes.

pXC5-$chvI_{D52A}$
chvI phospho-ablative mutant was amplified from the $chvI_{D52A}$ mutant gDNA by PCR primers 2405/2406. The PCR was then digested with *Nde* I and *Kpn* I, and ligated into the pXC-5 vector cut with the same restriction enzymes.

pXC5-$chvI_{D52E}$
chvI phospho-mimetic mutant was amplified from the $chvI_{D52E}$ mutant gDNA by PCR primers 2405/2406. The PCR was then digested with *Nde* I and *Kpn* I, and ligated into the pXC-5 vector cut with the same restriction enzymes.

pXGFPC-2-*chvI*
chvI was amplified from WT gDNA by PCR primers 2157/2411. The PCR was then digested with *Nde* I and *Kpn I*, and ligated into the pXGFPC-2 vector cut with the same restriction enzymes.

pXGFPC-2-*chvG*
chvG was amplified from WT gDNA by PCR primers 2155/2568. The PCR was then digested with *Nde* I and *Eco* RI, and ligated into the pXGFPC-2 vector cut with the same restriction enzymes.

pXGFPN-2-*chvG*
chvG was amplified from WT gDNA by PCR with primers 2569/2156. The PCR were then digested with *Kpn* I and *Sac* I and ligated into the pXGFPN-2 vector cut with the same restriction enzymes.

pXGFPC-2-$chvG_{H309N}$
chvG was amplified from $chvG_{H309N}$ gDNA by PCR primers 2155/2568. The PCR was then digested with *Nde* I and *Eco* RI, and ligated into the pXGFPC-2 vector cut with the same restriction enzymes.

pXCHYC-5-*chvG*
chvG was amplified from WT gDNA by PCR primers 2155/2568. The PCR was then digested with *Nde* I and *Eco* RI, and ligated into the pXCHYC-5 vector cut with the same restriction enzymes

pXCHYC-5-$chvG_{1\text{-}274}$
$ChvG_{1\text{-}274}$ was amplified from WT gDNA by PCR primers 2155/3131. The PCR was then digested with *Nde* I and *Eco* RI, and ligated into the pXCHYC-5 vector cut with the same restriction enzymes.

pXCHYC-5-$chvG_{273\text{-}534}$
$chvG_{273\text{-}534}$ was amplified from WT gDNA by PCR primers 3132/2568. The PCR was then digested with *Nde* I and *Eco* RI, and ligated into the pXCHYC-5 vector cut with the same restriction enzymes.

pXCHYC-5-$chvG_{1\text{-}114}$
$chvG_{1\text{-}114}$ was amplified from WT gDNA by PCR primers 2155/3133. The PCR was then digested with *Nde* I and *Eco* RI, and ligated into the pXCHYC-5 vector cut with the same restriction enzymes.

pMR15-P*chvI*
P*chvI* was amplified from WT gDNA by PCR with primers 3170/3171. The The PCR product was then digested with *Xba* I/*Xho* I and ligated into the pMR15 vector cut with the same restriction enzymes.

pMR15-P*dipM*
P*dipM* was amplified from WT gDNA by PCR with primers 3174/3175. The The PCR product was then digested with *Xba* I/*Xho* I and ligated into the pMR15 vector cut with the same restriction enzymes.

pMR15-P*ftsN*
P*ftsN* was amplified from WT gDNA by PCR with primers 3176/3177. The The PCR product was then digested with *Xba* I/*Xho* I and ligated into the pMR15 vector cut with the same restriction enzymes

pMR15-P*sigT*

P*sigT* was amplified from WT gDNA by PCR with primers 3182/3183. The PCR product was then digested with *Xba* I/*Xho* I and ligated into the pMR15 vector cut with the same restriction enzymes

pMR15-P*phyR*

P*phyR* was amplified from WT gDNA by PCR with primers 3184/3185. The PCR product was then digested with *Xba* I/*Xho* I and ligated into the pMR15 vector cut with the same restriction enzymes.

pET-28a-*chvI*

chvI (*CCNA_00237*) was amplified from WT gDNA by PCR with primers 2157/2158 (~710 bp), digested respectively with *Nde* I and *Sac* I, ligated into the pET-28a vector cut with the same restriction enzymes.
