# Supplementary material for: The two-component system ChvGI maintains cell envelope homeostasis in Caulobacter crescentus
Source: PLoS Genet. 2022 Dec 8;18(12):e1010465. doi: 10.1371/journal.pgen.1010465 (PMC9731502; doi:10.1371/journal.pgen.1010465)
Supplement: Attachment — Submitted filename: Response to referees 1.3.pdf [file pgen.1010465.s016.pdf]

Response to reviewers

Manuscript number : PGENETICS-D-22-00606

Title : **The two-component system ChvGI maintains cell envelope homeostasis in *Caulobacter crescentus***

Authors : Alex Quintero-Yanes[1], Aurélie Mayard[1] and Régis Hallez

Corresponding author : Régis Hallez

First of all, we want to thank the editor and the reviewers for your feedback on our manuscript. Your input helped us to understand that there are ways to improve the presentation of our results. Please find below a point-by-point response to each reviewer. To facilitate the comparison between the submitted and revised versions and to track changes made in the manuscript, we used green characters everywhere we modified the original text.

Reviewer #1

Major comments

1. The authors use different types of osmotic stress to activate the ChvGI system for different experiments. However, it is not obvious whether each causes an equivalent degree of osmotic stress or whether some are more "stressful" than others. Given that the data indicates that sigT and ChvGI are activated at different "levels" of osmotic stress, and that they antagonize each other, it would reinforce the paper's conclusions to include a calibration experiment where delta-chvI viability and/or ChvI or SigT-dependent promoter activation in response to the different osmotic stresses used are assessed in parallel. This would be particularly relevant for comparing M2G and PYE + 6% sucrose, since the ChIP-Seq experiment was performed in M2G while the RNA-Seq experiment was performed in PYE + sucrose.

Our response: As suggested by the reviewer #1, we included calibration experiments in the revised version. First, we measured the activity of three ChvI-dependent promoters (P_{dipM}, P_{ftsN} and P_{phyR}) in WT cells exposed to different osmotic stresses (Figure S2A-C and lines 215-223). Then, we measured the activity of *chvI* promoter upon different osmotic stresses in WT, Δ*chvI* and Δ*sigT* backgrounds (Figure S3A and lines 234-245). Finally, we evaluated viability of WT, Δ*chvI*, Δ*sigT* and Δ*chvI*Δ*sigT* cells on plates supplemented with different concentrations of different osmolytes (Figure S3B and lines 246-254). These calibration experiments showed that ChvI-dependent promoters gradually increased their activity proportionally to the concentration of osmolytes with M2G having the strongest inducing effect. Together, this confirms that SigT and ChvGI are activated at different "levels" of osmotic stress.

2. In Figure 3, beta-galactosidase reporter activity data are presented to confirm the results of the ChIP-Seq and RNA-Seq data, performed under the same osmotic stress conditions (3B, matches ChIP-Seq, 4 hours in M2G medium; Figure 3D, matches RNA-Seq, 4 hours in PYE + 6% sucrose). However, for some of the promoters, the measured activity in all strains shows large differences between these two conditions.

The chvI promoter activity is similar in both conditions, but the dipM, ftsN and phyR promoter constructs have much lower values, by approximately a factor of 10, in PYE + 6% sucrose compared to M2G. While it doesn't invalidate the authors' conclusion that ChvI can act as both a positive and a negative regulator of gene expression, it does indicate that (1) these genes respond differently to these environmental conditions, potentially through other factors that regulate their expression, and (2) that these osmotic stress inducing conditions are not fully equivalent to each other. In particular, M2G-induced starvation will also lead to alterations in cell cycle gene regulation, which PYE + sucrose would not do, and these alterations would very likely overlap with ChvI-dependent regulation of cell division genes. This should be acknowledged in the main text.

Our response: To clarify this point, we performed β-gal assays for *dipM*, *ftsN* and *phyR* promoters in WT cells exposed to different osmotic conditions, including the ones used for the RNA-seq and ChIP-seq experiments. As explained above, their activity gradually increased proportionally to the concentration of osmolytes while M2G had the strongest inducing effect (Figure S2A-C and lines 215-223). We also mentioned that in addition to osmolality, the transition from complex medium (PYE) to synthetic minimal medium (M2G) likely increases the stress sensed by the cells (lines 221-223).

3. Lines 191-194, can the authors elaborate on how SigT might act as a negative regulator of ChvI transcription, given that it is an alternative sigma factor and therefore direct regulation by SigT would normally be positive?

Our response: Considering that SigT is a sigma factor, it is indeed expected that SigT controls *chvI* expression indirectly. One possibility would be that SigT activates the transcription of a negative regulator(s) of the *chvIG-hprK* operon. Previously, the SigT regulon was determined using RNA-seq in hyperosmotic conditions and putative transcriptional repressors of the AcoR, TetR and MarR-repressor families, such as CCNA_03694, CCNA_00967 and CCNA_03426, respectively, were significantly downregulated in a Δ*sigT* mutant. However, we decided to not include a discussion/speculation about how SigT might negatively regulate ChvI since in this manuscript, we describe the role of and the regulations mediated by ChvGI.

Minor comments

line 53, replace "his" with "its"

Our response: Amended (line 53).

line 156, not sure what is meant by "additional" here? Additional to what?

Our response: To make the statement clear to the reader, we changed the sentence to " *These data indicate that additional cell envelope defects to those caused by chvT and ntrX dysregulation in ΔchvI mutants are responsible for growth impairment on hypertonic conditions* (lines 158-159).

line 163, replace "nonetheless" with "however"

Our response: Amended (line 165).

line 267, associated "with" (not "to")

Our response: Amended (line 289).

Figures 4D/5F and legend, indicate how the quantified values in the graphs were derived from the band intensities (either here or in methods).

Our response: The quantification methodology for ChvI phosphorylation is now included in the methods section (lines 754-764).

Reviewer #2

Major comments

This study adds significantly to the understanding of the ChvGI regulators in C. crescentus. The experiments are wide-ranging and generate a wealth of data, that expands on prior studies in C. crescentus.

The introduction does a nice job of succinctly providing context for the study, however I find that the transcriptome analysis does not take advantage of prior studies in other APB to compare the Cc regulon to those of other systems. The ChvG-ChvI (ExoR) transcriptomic data is available for S. meliloti and A. tumefaciens and could be productively compared. These systems are activated by acidic pH through proteolytic cleavage of the ExoR protein and it is a shame not to compare and contrast the Cc system in which no such regulator has been identified.

Our response: As suggested by the reviewer #2, we included in the Discussion section a comparison between Chv systems in alpha-Proteobacteria (lines 401-410). To facilitate these comparisons, the differentially regulated genes found in our RNA-seq data were categorised by function with the database of clusters of orthologs groups (COG) (Figure 3C, lines 202-209).

I also find the comparison between the ChIP-Seq and RNASeq within this study to be quite minimal. How strong is the agreement? How much of the ChvGI regulon is directly vs indirectly impacted?

Our response: We included information on the percentage of genes up- and down-regulated in the RNA-seq that were identified in the ChIP-seq (line 186). Also, we used promoter sequences of genes (i) found in both the ChIP-seq and RNA-seq data and (ii) whose expression was shown to be ChvI-dependent to find a putative consensus binding sequence using MEME (Figure 3E, lines 224-229).

In addition, it's important to note that we used different growth conditions for ChIP-seq (M2G) and RNA-seq (PYE + 6% sucrose). The reason is simply because the Δ*chvI*

mutant, which was required for the RNA-seq experiments, could not grow in M2G in contrast to the WT. To circumvent this problem, the Δ*chvI* mutant was grown in PYE and then exposed to 6% sucrose for 4 hours before extracting RNA samples. Thus, further describing the overlap between the ChIP-seq and RNA-seq data by the difference in the composition and the osmotic imbalance of these two growth conditions is a bit difficult. Nevertheless, despite this difference, we could find a large fraction (42%) of peaks identified in the ChIP-seq as positively regulated in the RNA-seq data.

Overall, the fold-changes of most target genes and the impacts of the regulators seem modest, often less than two-fold for the beta-gal fusions. Is there any explanation why this might be?

Caulobacter has a biphasic life cycle so perhaps expression is biased towards one cell type and the total amount of expression normalized to total biomass is an underrepresentation? Even so, this would likely only have a two-fold effect at most. How do these levels compare to other regulatory systems in Cc?

Our response: First of all, a small impact on promoter activity (β-gal assays) could have a strong effect on mRNA levels, in particular if the corresponding mRNAs are produced at low levels and/or are unstable. In support of this, according to our RNA-seq data, at least 355 genes are up/down regulated in Δ*chvI vs* WT with a FC≥2-fold, which represents around the 9% of the genome. Also, 58 genes are upregulated with a FC≥5-fold, which cannot be considered as modest. On the other hand, by comparing our RNA-seq data, generated from Δ*chvI* and WT cells grown in PYE and exposed to 6% sucrose for 4 hours, to the one recently published (Stein *et al.,* 2021), generated from the Δ*chvI* mutant overexpressing a phosphomimetic variant $chvI_{D52E}^{++}$ grown in PYE, we have a large overlap between these two sets of data.

A potential bias towards one cell type is very unlikely since we did not detect cell cycle defects in Δ*chvI* in comparison to WT. As shown below, the proportion of G1cells as determined by FACS was similar in both WT and Δ*chvI* cells grown in PYE and exposed to 6% sucrose for 4 hours, that are the conditions used for the RNA-seq.

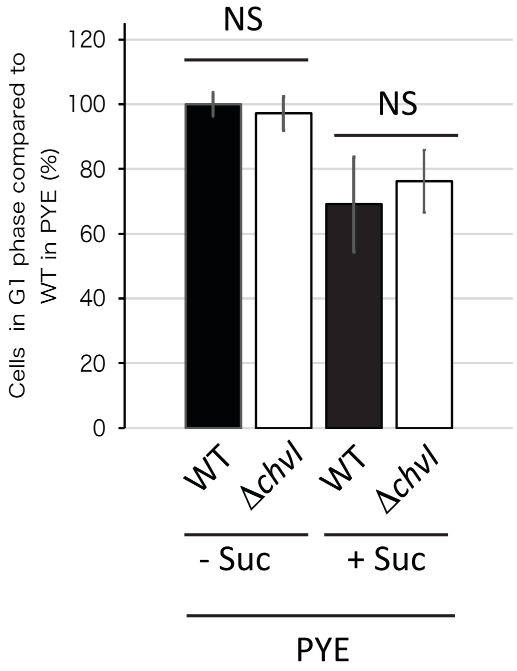

Fluorescence-activated cell sorting of WT and Δ*chvI* cells grown in PYE and exposed for 4 hrs to 6% sucrose. Annova single factor comparison between WT and Δ*chvI* in PYE without sucrose (-suc) data *P*- value: 0.475 *F crit* 7.708 and between WT and Δ*chvI* in PYE with sucrose (+ suc) data *P*- value: 0.107 *F crit* 7.708 and NS = $p > 0.05$

Stein BJ, Fiebig A, Crosson S. The ChvG-ChvI and NtrY-NtrX two-component systems coordinately regulate growth of Caulobacter crescentus. Journal of bacteriology. 2021 Aug 9;203(17):e00199-21.

Minor Comments:

1. L53 – "its" rather than his

Our response: Amended (line 53).

2. L70-1. The proteolysis of ExoR was also reported for S. meliloti – Lu et al. 2012, JB 194:4029. In fact, the proteolysis was reported first in S. meliloti and cited in the Wu et al. paper.

Our response: Amended (lines 70-72).

3. L106. "We provide a ChvI regulon…" should be "We provide information on the ChvI regulon…." Or we elucidate the……" ChvI regolun

Our response: Amended (line 107).

4. L146. This is PYE + 50 mm KCl in the figure.

Our response: Amended (line 149).

5. L207-8. Why were the conditions in PYE modified from those used in the expression and resistance assays to PYE + 6% sucrose. Some explanation of this shift is warranted.

Our response: As explained above, the reason for this change is simply because the Δ*chvI* mutant cannot grow in M2G in contrast to the WT. To circumvent this problem, the Δ*chvI* mutant was grown in PYE and then exposed to 6% sucrose for 4 hours before extracting RNA samples. A brief explanation on the use of PYE + 6% sucrose is now included (line 182).

6. L213-4. Is there any explanation for the incongruity between the RNA-Seq and the fusions for ftsN and phyR?

Our response: We showed that the osmotic stress used for RNA-seq (PYE + 6% sucrose) has lower impacts on *dipM*, *ftsN* and *phyR* promoter activity than M2G. It is possible that differences in mRNA levels in these mild stressful conditions were too weak to be detected in our RNA-seq experiment. It's noteworthy that none of these 3 genes (*dipM, ftsN* and *phyR*) was detected in the RNA-seq performed with the phosphomimetic $chvI_{D52E}^{++}$ (Stein *et al.,* 2021). It has to be considered that there are limitations to each technique, including RNA-seq (Liu *et al.,* 2016), and that combining

transcriptomic approaches, as we did in our study, is a good approach to unveil more candidates.

Stein BJ, Fiebig A, Crosson S. The ChvG-ChvI and NtrY-NtrX two-component systems coordinately regulate growth of Caulobacter crescentus. Journal of bacteriology. 2021 Aug 9;203(17):e00199-21.

Liu P, Sanalkumar R, Bresnick EH, Keleş S, Dewey CN. Integrative analysis with ChIP-seq advances the limits of transcript quantification from RNA-seq. Genome research. 2016 Aug 1;26(8):1124-33.

7. L229. AND ALSO THOSE ENCODING the elongasome components…– otherwise, this can be interpreted as other genes/proteins affected by StaR.

Our response: Amended (line 195).

8. L248-9. Given that it was the chvG mutant that had to be tested for chvI phosphorylation it would be more appropriate to document the growth of the chvG mutant in the M5GG in 4A. Was the chvG growth phenotype identical to that of the chvI mutant?

Our response: As requested by the reviewer #2, we now included growth curves of Δ*chvG* mutant grown in M5GG (Figure 4A and line 265). These data show that both Δ*chvI* and Δ*chvG* mutants grew similarly to WT in M5GG.

9. L250-53. It needs to be made clear here that these results are immunoprecipitation reactions with anti-ChvI Abs. As described in the results this was quite unclear and I found myself questioning how these in vivo labeling reactions were so clean. Also should be mentioned in the legend for Fig. 4. Also, there is no internal control and with limited details on how these were normalized. For an example where this could be problematic, the ChvI-P in the wild type with no sucrose in panels B and C is not comparable in intensity.

Our response: As requested by the reviewer #2, we now included details about how ChvI was pulled-down and how the phosphorylation assays were performed (lines 268-

270 and 272-273). *In vivo* phosphorylation assays are normalized to the protein levels. However, since we worked with γ-[^{32}P]ATP radioactive samples, we could not do normalization directly on the samples used for phosphorylation assays. Instead, we independently measured the ChvI protein content by Western Blot after treatment with sucrose and antibiotics in the exact same conditions (7 min exposure) but without γ-[^{32}P]ATP, and we did not observe significant differences in protein abundance (please see image below).

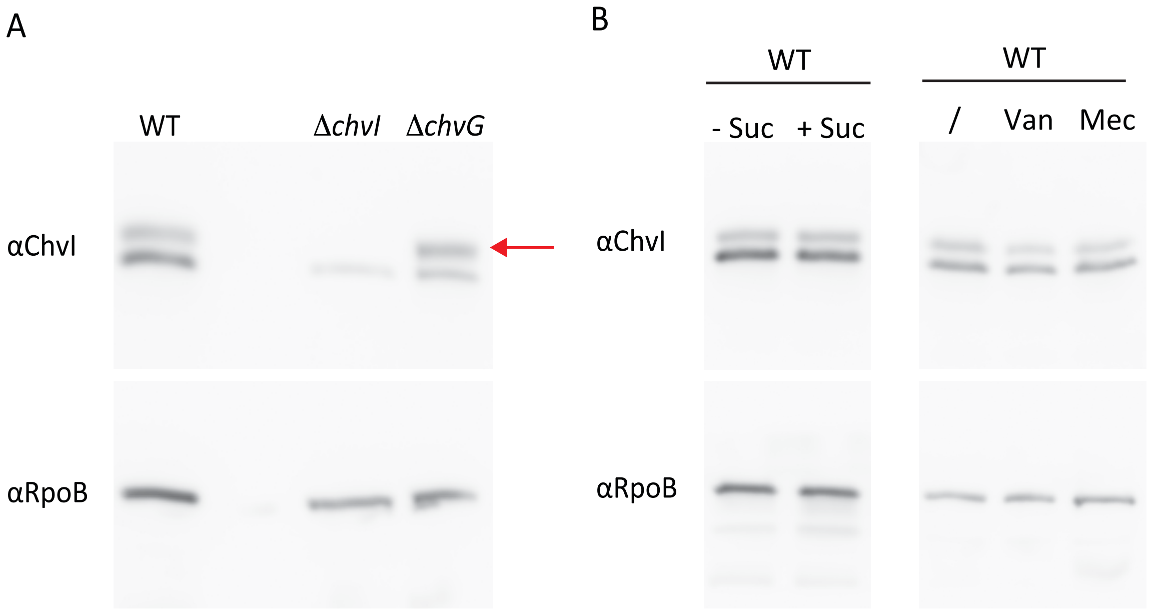

Western blot analysis with anti-ChvI antibodies of protein samples from WT, Δ*chvI* and Δ*chvG* grown in (A) M5GG (without stress) and (B) WT samples treated for 7 min with either 6% sucrose (+ Suc), 10 μg ml^{-1} vancomycin and 100 μg ml^{-1} mecillinam. Red arrow points out to the band corresponding to ChvI. Anti-RpoB antibodies were used as loading control.

10. L266. The polymyxin B treatment seems to be right on the edge of significance. There does seem to be a consistent difference in growth.

Our response: There is indeed some difference in growth between WT and Δ*chvI* when exposed to polymyxin B. However, we did not find correlation between growth and survival. In addition, there is a high variability on growth in the presence of

polymyxin B for both strains. We included these observations in the revised manuscript (lines 290-295).

11. L283-5. The difference in timing would be more convincing presented as a time course.

Our response: As suggested by the reviewer #2, we performed a time course of *chvI* promoter activity upon vancomycin or mecillinam treatment. However, as illustrated below, we observed that mecillinam treatment already increased P_{chvI} activity in a similar time window than vancomycin exposure although the timepoint 1 hr is only statistically significant for vancomycin. Therefore, the timing of *chvI* expression does not support anymore our statement, that the vancomycin and mecillinam treatments could require different exposure times to reach similar ChvGI activation. For that reason, we decided to remove this graph from the manuscript since it was added only for that purpose. However, if requested by the editor, we could include this graph as supplementary data to show that the induction timing of P_{chvI} activity is similar between both antibiotic treatments. It's noteworthy that the time of antibiotics exposure for the phosphorylation assay (7 minutes) is much shorter than for the β-gal asays (1 hr). Thus, we still discuss the possibility that there could be different time windows of vancomycin and mecillinam treatment to activate ChvG phosphorylation (lines 305-309, lines 477-479).

12. L338. In C. crescentus.

Our response: Amended (line 338).

13. L339. Was there any logic to why the sensor domain was truncated at pos. 114? Seems an odd truncation site.

Our response: We understand it seems odd using this residue without context. In another project unrelated to the current manuscript, we performed a genetic screen in which a point mutation at that position in ChvG was identified. We do not yet

understand why this mutation came out in our screen. We believe it would be confusing for the readers to describe this genetic screen and the logic behind using this truncated variant. Given that (i) the truncated variant at position 114 does not bring new data in comparison to the two other truncations (1-202 and 1-274) and (ii) no other data we generated so far with this mutant in the other project can be connected to the current manuscript, we decided to remove data about position 114 from Figures 7 and S5. However, if requested by the editor, we could include these data in the manuscript as it was initially submitted.

14. L401. OF the SigT-dependent regulon

Our response: Amended (line 442).

15. L456. How do the authors propose that ChvG senses the pressure? Presumably they mean osmotic pressure. Are there comparable systems in other bacteria. Would this be similar to EnvZ-dependent sensing in E. coli?

Our response: Yes, we meant osmotic pressure, this has been amended (line 500). As far as we know, EnvZ responds to cytoplasmic signals generated by changes in the extracellular osmolyte concentrations. In our case, the periplasmic domain and the transmembrane helices seem to be important, suggesting that it is rather a change in the periplasm or in the membrane that is sensed. This could be for instance a rapid change in PG synthesis or modification that would happen upon osmotic upshift. Speculative mechanisms of ChvG sensing and activation are discussed (lines 498-505).

16. L471. Should provide a reference for the statement about MreB orthologs.

Our response: Amended (line 515).

17. L493-4. This reads confusingly – Perhaps – "..under conditions in which the cell envelope is unperturbed compared to conditions which compromise the envelope."?

Our response: This has been clarified in the revised manuscript (line 537-540).

18. L982. How are these extracts normalized?

Our response: As already explained in comment 9, *in vivo* phosphorylation assays are normalized to the protein levels but since we worked with γ-[^{32}P]ATP radioactive samples, we could not do normalization directly on the samples used for phosphorylation assays. Instead, we independently measured the ChvI protein content by Western Blot after treatment with sucrose and antibiotics in the exact same conditions (7 min exposure) but without γ-[^{32}P]ATP, and we did not observe significant differences in protein abundance.

Reviewer #3:

Major suggestion/concerns

Were the peak regions identified by ChIP-seq analyzed for a consensus sequence that may represent the ChvI binding site and if putative binding site sequences can be identified, is there a correlation between positive or negative regulation by ChvI with the position of the binding site relative to promoter motifs and transcription start sites?

Our response: As suggested by the reviewer #2, promoter sequences of genes (i) found in both the ChIP-seq and RNA-seq data and (ii) whose expression was shown to be ChvI-dependent were used to find a putative consensus binding sequence using MEME (Figure 3E, lines 224-229). This motif was identified upstream of the TSS of 18 genes, 12 positively and 6 negatively regulated by ChvI (**See figure below**). For most of the positively-regulated genes, the motif was located upstream of the -10 position related to the TSS while it was found downstream of the TSS of all the negatively-regulated genes. However, for the positively-regulated genes, the position of the boxes is not systematically found upstream of the -35 on the + strand, which is expected for a transcriptional activator that interacts with the RNAP. For that reason, we did not

include this figure in the revised manuscript. But if requested by the editor, we could add this figure as supplementary data as well as a short description of our observations.

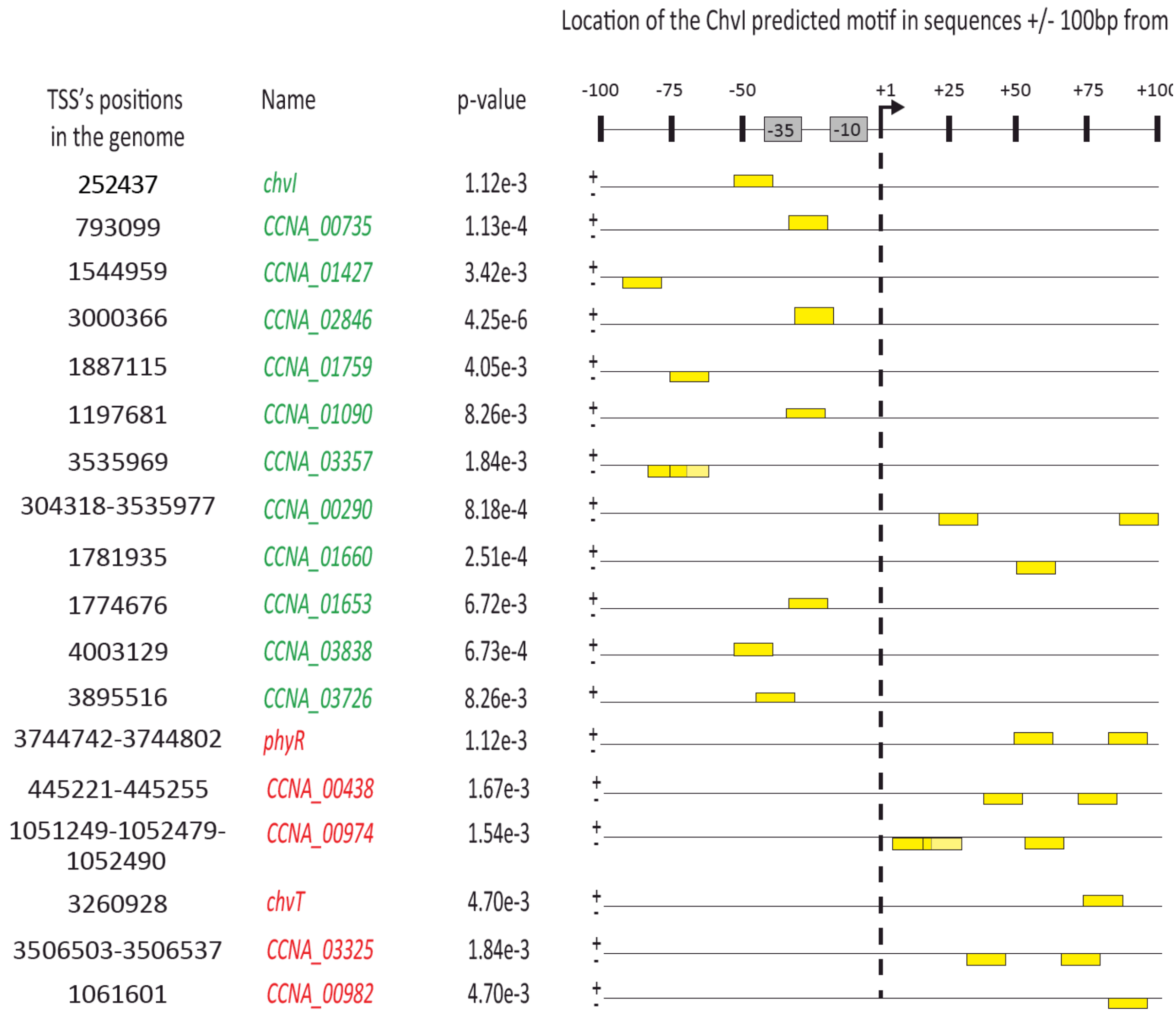

Localisation of ChvI consensus sequences. Position and orientation of the motifs (yellow boxes) relative to the TSS (+1) and transcription direction (+ / -) of genes identified in the ChIP-seq, and found to be either positively (green) or negatively (red) regulated by ChvI. Two or more motifs per gene indicate that the gene has more than one TSS and that the motif was found in the vicinity of them.

Fig. 4B and C, 5F: Was a loading control used to guarantee that the same amount of protein was loaded? This is considered important for the quantitative comparisons.

Our response: Yes indeed, we measured the ChvI protein content by Western Blot after treatment with sucrose and antibiotics in the same conditions used for

phosphorylation assays (growth in PYE, then 7 min exposure to stress) but without γ-[^{32}P]ATP. As shown in the figure below, we did not detect significant differences in protein abundance (see image below).

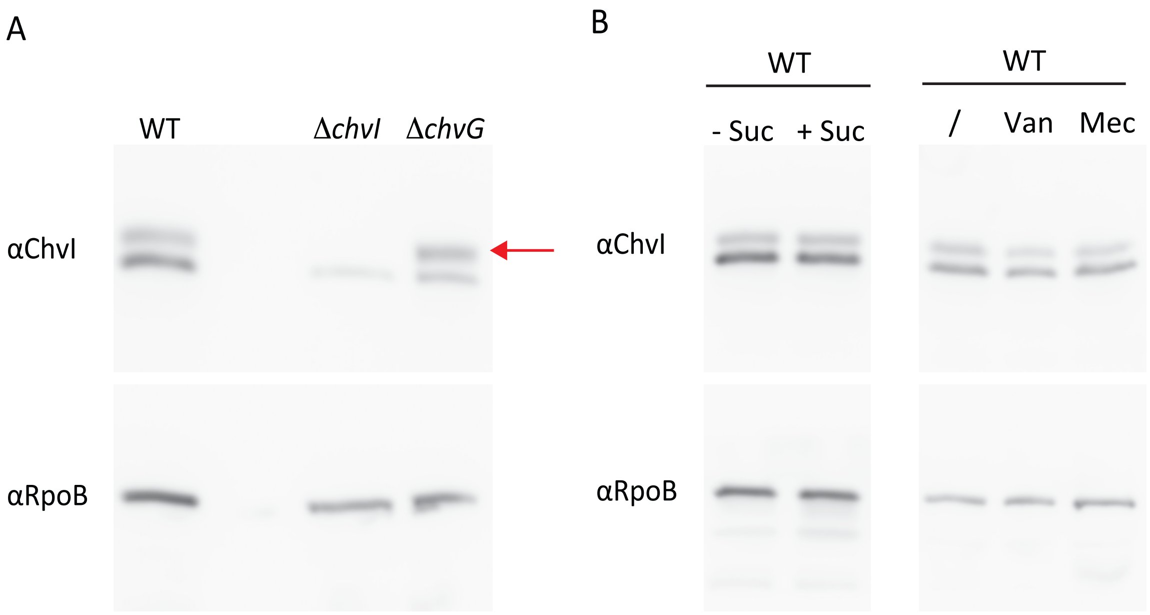

Western blot analysis with anti-ChvI antibodies of protein samples from WT, Δ*chvI* and Δ*chvG* grown in (A) M5GG (without stress) and (B) WT samples treated for 7 min with either 6% sucrose (+ Suc), 10 μg ml^{-1} vancomycin and 100 μg ml^{-1} mecillinam. Red arrow points out to the band corresponding to ChvI. Anti-RpoB antibodies were used as loading control.

Minor comments

l. 67-68: "pathogenic regulator" should be replaced by 'pathogenicity regulator' or regulator of pathogenicity"

Our response: Amended (line 67).

l. 136 "… whereas expressing back chvI" Please rephrase.

Our response: Amended (line 137).

ll. 137-138: Please add references for the phosphor-mimetic and phosphor-ablative chvI variant, as a proof for the properties of these protein variants is important, but provided in a previous study.

Our response: Amended (line 138).

ll. 166-168: "This suggests either that ChvID52E does not perfectly mimic phospohorylated …" Yet another possibility is that the level of active ChvI is too high, as every protein of the phosphor-mimetic variant is probably active and cannot be inactivated by dephosphorylation.

Our response: Yes indeed, this is why we mention that unphosphorylated ChvI might also be required for optimal growth (line 170). We also discuss about how constitutive active stress response systems can be detrimental in bacteria (lines 457-470).

ll. 191-192: "… the chvI promoter (PchvI) was significantly higher …" should read ""… the chvI promoter (PchvI) activity was significantly higher …"

Our response: Amended (line 241).

ll. 196-198: "To better understand the regulatory mechanism between ChvI and SigT we tested viability of mutants for the genes in osmotic conditions used in this study, …" Please rephrase for clarity. It is not clear what is meant by "mutants for genes in osmotic conditions".

Our response: This has been rephrased (lines 246-249).

l. 198, l. 200: "… that is PYE supplemented …" should read "… that is PYE supplemented with …"

Our response: Amended (line 248).

ll. 202-203: "… the viability of ΔsigT declined." should read "… the viability of ΔsigT mutant declined."

Our response: Amended (line 251).

ll. 216-217: "… dipM and chvI promoters activity …" should read "… dipM and chvI promoter activity …"

Our response: Amended (line 215).

ll. 245-246: "… which is much less that in M2G …" should read "… which is much less than in M2G …"

Our response: Amended (line 264).

Fig. 1A, Fig. 2, Fig. 5E: spotting assays: how many replicates are represented by the displayed images? Results from further replicates should be provided in a data repository.

Our response: As requested by the reviewer#3, we now included the replicates as supplementary data.

Fig. 1B-D, Fig. 4A, Fig. 5A-D: y-axis – replace commas used as decimal separator

Our response: Amended.

Fig. 2: The two further replicates not shown should be included in the supplement or in a data repository.

Our response: As stated above, we now included the replicates as supplementary data.

References in reference list require further formatting.

Our response: Amended.

Have all data underlying the figures and results presented in the manuscript been provided?

Large-scale datasets should be made available via a public repository as described in the *PLOS Genetics* data availability policy, and numerical data that underlies graphs or summary statistics should be provided in spreadsheet form as supporting information.

Reviewer #2: **No:** Data for most graphs was not included. Also, the gel for 32P labeling experiment was highly cropped and the original was not provided as back-up. Large scale data was deposited in appropriate repositories.

Our response: Please find below uncropped images for 32P labelling experiments. These uncropped images have been made available as supplementary data together with the replicates.

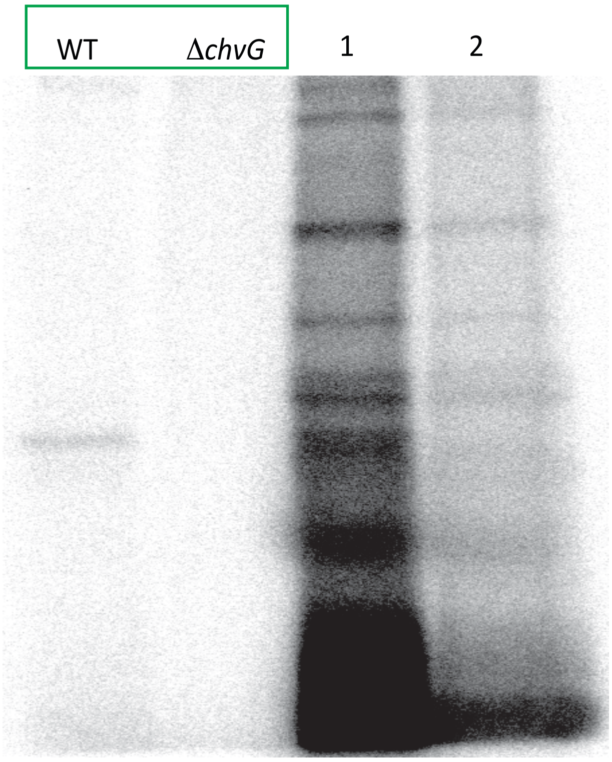

Original image for gel presented in Fig. 4B. Lanes corresponding to strains presented in Fig. 4B (WT and Δ*chvG*) are highlighted in green square. Lanes 3 and 4 correspond to WT and Δ*chvG* samples, respectively, from early setup experiments designed to treat cells with excess (in this case with 50 μg ml^{-1}) of vancomycin.

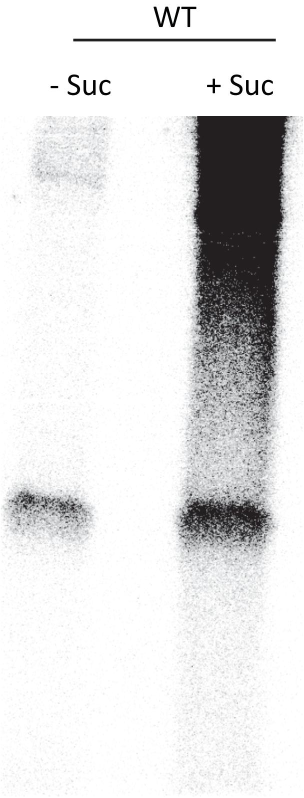

Original image for gel presented in Fig. 4C.

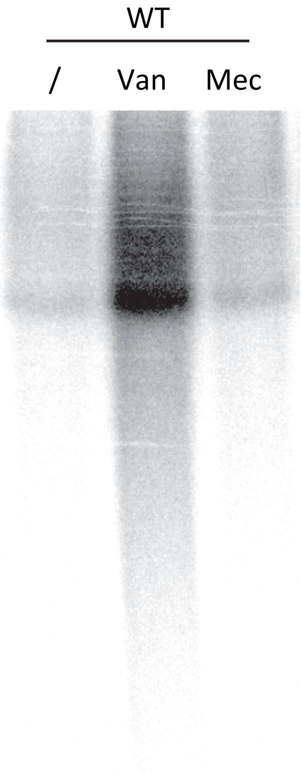

Original image for gel presented in Fig. 5F. We would like to clarify that the bands look higher compared to the two previous gels because the original SDS-PAGE gel was cut in the upper part to improve detection of the signal in ChvI by avoiding the exposure of the smear in the upper part of the gels (as seen in the original image for gel in Fig. 4C) to the phosphoscreen.
