# Supplementary material for: The two-component system ChvGI maintains cell envelope homeostasis in Caulobacter crescentus
Source: PLoS Genet. 2022 Dec 8;18(12):e1010465. doi: 10.1371/journal.pgen.1010465 (PMC9731502; doi:10.1371/journal.pgen.1010465)
Supplement: Attachment — Submitted filename: Response to referees 2.1.pdf [file pgen.1010465.s017.pdf]

Response to reviewers

Manuscript number: PGENETICS-D-22-00606R1

Title: **The two-component system ChvGI maintains cell envelope homeostasis in *Caulobacter crescentus***

Authors: Alex Quintero-Yanes[1], Aurélie Mayard[1] and Régis Hallez

Corresponding author: Régis Hallez

Please find below a point-by-point response to each reviewer. To track changes made in the manuscript, we used green characters everywhere we modified the original text.

Reviewer #1

The authors have made thorough revisions to their manuscript which address all my prior comments and experimentally strengthen the conclusions that they draw from the data. The manuscript is noticeably improved as a result. I have only a few minor comments on the revised version, below.

Minor comments

Line 128, for clarity: "viability of chvIG mutants on M2G plates lacking Na+ and K+ salts

Our response: Amended (line 127).

Line 212, remove "for"

Our response: Amended (line 211).

Lines 250-252, while it doesn't invalidate the authors conclusions, I don't really see much difference in viability between WT and the sigT mutant strain in any condition tested, including the most stressful ones (Fig S3B). In some of the replicates in the supplemental data it is clearer, but still weak. Suggest "the effect of these osmotic stressors on the sigT mutant was minimal" or similar phrasing. I don't think the authors need to make any more experiments, as the effect of sigT loss during osmotic stress has been characterized already in other work.

Our response: This has been rephrased as "… the viability of Δ*sigT* mutant was weakly impacted…" (line 251) and "Together, these data suggest that ChvGI is much more sensitive to osmotic stress than SigT" (lines 252-253)

Line 346, remove "to"

Our response: Amended (line 345).

Line 451, "higher salt concentrations" (remove extra "s")

Our response: Amended (line 450).

Line 479, based on the data presented, the authors haven't directly observed that the antibiotics cross the outer membrane at different rates, it is still a hypothesis. Suggest "deduce" instead of "observe" here.s

Our response: This has been rephrased as "This could be explained if these antibiotics cross the outer membrane with different efficiency. Consistent with this idea, our data suggest that vancomycin is efficiently transported by the TBDR ChvT, while other antibiotics …" (lines 477-479).

Reviewer #2

Quintero-Yanes et al. have extensively revised their manuscript from the prior submission, and have largely addressed my initial comments, and most of those from the other reviewers. For the most part the manuscript has been substantially improved.

The section on the identified ChvI binding site remains a little fuzzy, and could be cleared up. I think the authors should add the binding site map they present in their responses to the supplemental figures. Also, they should include the previously published sequence for the ChvI binding site from S. meliloti, so that their sequence from C. crescentus can be compared to what was proposed before. I am more inclined to believe the binding site identified here based on the ChIP-Seq analysi, not to mention that fact that they are comparing this to a different genus of bacteria in a different Order. I do agree that experimental validation is also needed from this analysis, but the current manuscript already represents a large study and I am not suggesting that this validation should be included here.

Our response: As suggested by the reviewer #2, we have now included the predicted binding site in *S. meliloti* in the discussion (line 414). However, we did not include the binding site map to not increase even more the fuzziness of this section. Indeed, as explained in our previous responses, the position of the boxes for the positively-regulated genes is not systematically found upstream of the -35 on the + strand, which is expected for a transcriptional activator that interacts with the RNAP.

There are few other minor issues in this section as well.

L414 - consensus ChvI bonding sequence should be "ChvI binding sequence"

Our response: Amended (line 414).

L416 - The phrase "....for the ChvI showed a reasonable conserved DNA binding motif..." is confusing. It sounds as if they have defined a DNA binding motif on ChvI. Rephrasing to read "...revealed reasonably conserved DNA sequences that could be recognized by the ChvI protein...."

Our response: Amended (lines 415-416).

Have all data underlying the figures and results presented in the manuscript been provided?

Large-scale datasets should be made available via a public repository as described in the *PLOS Genetics* data availability policy, and numerical data that underlies graphs or summary statistics should be provided in spreadsheet form as supporting information.

Reviewer #1: Yes

Reviewer #2: ***No:*** *The numerical data underlying information presented in graphical form has not been provided (or I could not find it), although the authors have uploaded large scale data to appropriate databases and now include full mages of their gels.*

Our response: Numerical data were already provided as Supplementary Figure 7 (line 1183).
