# Supplementary material for: The two-component system ChvGI maintains cell envelope homeostasis in Caulobacter crescentus
Source: PLoS Genet. 2022 Dec 8;18(12):e1010465. doi: 10.1371/journal.pgen.1010465 (PMC9731502; doi:10.1371/journal.pgen.1010465)
Supplement: Attachment — Submitted filename: PlosGen MS review 2022.docx [file pgen.1010465.s015.docx]

Review of “The two-component system ChvGI maintains cell envelope homeostasis in Caulobacter crescentus”

Summary

The manuscript describes the effect of osmotic stress on the two-component system ChvGI in Caulobacter crescentus. This system has been extensively studied in other alpha-proteobacteria, and more recently in Caulobacter as well (references 9, 11 and 12). These studies showed that, in contrast to the symbiotic or pathogenic alpha-proteobacteria in which ChvGI appeared to be primarily a sensor of host cell association, in Caulobacter it is a sensor of antibiotic-induced cell envelope stress (reference 12) and a regulator of growth in minimal medium (reference 9). This manuscript builds on those studies, in showing that the primary factor which stimulates ChvGI activation is osmotic stress, which can be induced in minimal medium (M2G), or in PYE supplemented with sucrose, two different concentrations of NaCl, or KCl. Moreover, the system is also activated by antibiotics which interfere with peptidoglycan synthesis, and the direct regulon of ChvI (as defined by ChIP-Seq and confirmed by RNA-Seq and promoter activity assays) includes many genes involved in cell envelope homeostasis (in particular peptidoglycan synthesis and remodeling, outer membrane proteins and cell division proteins). Hence, the poor growth of chvI mutants in M2G minimal medium is due to the inability to respond appropriately to the cell envelope stress imposed by the higher osmotic strength of minimal medium compared to PYE. ChvGI responds to lower levels of osmotic stress than the alternative sigma factor SigT, which had previously been characterized as an important regulator of the osmotic stress response in Caulobacter, and regulation of sigT and chvIG is antagonistically linked. Using fluorescence microscopy, the authors also show that the ChvG sensor kinase has an unusual subcellular localization pattern more typically seen for peptidoglycan-synthetic enzymes, and that this changes upon osmotic stress from a patchy-spotty localization around the cell periphery, to a sharply defined focus at midcell.

The major strength of this manuscript is that it formally defines ChvGI as an osmotic stress-sensing two-component system of the alpha-proteobacteria, which had been previously unknown to the field since the alpha-proteobacteria do not share orthologues of other envelope stress-sensing two-component systems (eg. the Cpx system) found in other classes of bacteria. Moreover, the finding that ChvG shares a common localization pattern with components of the peptidoglycan synthetic machinery, including a localization shift upon exposure to osmotic stress, is highly novel. It likely points towards a direct role of the system in sensing peptidoglycan integrity, potentially relevant to other classes of bacterial sensor kinases which sense cell envelope stress. This has not yet been studied in depth, but it can be seen in the previous study on high-throughput protein localization in Caulobacter (reference 24) that some, but not all, of the other annotated sensor kinase proteins also display (in absence of stress) this localization pattern. This is likely to be an important future research field in bacterial cell biology. The data presented on the antagonistic regulation of sigT by ChvI and vice versa also adds significantly to our understanding of the general stress response in the alpha-proteobacteria.

Major comments

1. The authors use different types of osmotic stress to activate the ChvGI system for different experiments. However, it is not obvious whether each causes an equivalent degree of osmotic stress or whether some are more “stressful” than others. Given that the data indicates that sigT and ChvGI are activated at different “levels” of osmotic stress, and that they antagonize each other, it would reinforce the paper’s conclusions to include a calibration experiment where delta-chvI viability and/or ChvI or SigT-dependent promoter activation in response to the different osmotic stresses used are assessed in parallel. This would be particularly relevant for comparing M2G and PYE + 6% sucrose, since the ChIP-Seq experiment was performed in M2G while the RNA-Seq experiment was performed in PYE + sucrose.

2. In Figure 3, beta-galactosidase reporter activity data are presented to confirm the results of the ChIP-Seq and RNA-Seq data, performed under the same osmotic stress conditions (3B, matches ChIP-Seq, 4 hours in M2G medium; Figure 3D, matches RNA-Seq, 4 hours in PYE + 6% sucrose). However, for some of the promoters, the measured activity in all strains shows large differences between these two conditions. The chvI promoter activity is similar in both conditions, but the dipM, ftsN and phyR promoter constructs have much lower values, by approximately a factor of 10, in PYE + 6% sucrose compared to M2G. While it doesn’t invalidate the authors’ conclusion that ChvI can act as both a positive and a negative regulator of gene expression, it does indicate that (1) these genes respond differently to these environmental conditions, potentially through other factors that regulate their expression, and (2) that these osmotic stress inducing conditions are not fully equivalent to each other. In particular, M2G-induced starvation will also lead to alterations in cell cycle gene regulation, which PYE + sucrose would not do, and these alterations would very likely overlap with ChvI-dependent regulation of cell division genes. This should be acknowledged in the main text.

3. Lines 191-194, can the authors elaborate on how SigT might act as a negative regulator of ChvI transcription, given that it is an alternative sigma factor and therefore direct regulation by SigT would normally be positive?

Minor comments

line 53, replace “his” with “its”

line 156, not sure what is meant by “additional” here? Additional to what?

line 163, replace “nonetheless” with “however”

line 267, associated “with” (not “to”)

Figures 4D/5F and legend, indicate how the quantified values in the graphs were derived from the band intensities (either here or in methods)
